# Characterization of a [4Fe-4S]-dependent LarE sulfur insertase that facilitates nickel-pincer nucleotide cofactor biosynthesis in *Thermotoga maritima*

**DOI:** 10.1016/j.jbc.2022.102131

**Published:** 2022-06-11

**Authors:** Shramana Chatterjee, Kristine F. Parson, Brandon T. Ruotolo, John McCracken, Jian Hu, Robert P. Hausinger

**Affiliations:** 1 Department of Microbiology and Molecular Genetics, Michigan State University, East Lansing, Michigan, USA; 2 Department of Chemistry, University of Michigan, Ann Arbor, Michigan, USA; 3 Department of Chemistry, Michigan State University, East Lansing, Michigan, USA; 4 Department of Biochemistry and Molecular Biology, Michigan State University, East Lansing, Michigan, USA

**Keywords:** iron-sulfur protein, sulfotransferase, nickel, enzyme, biosynthesis, cofactor, pincer, βME, 2-mercaptoethanol, CID, collision-induced dissociation, EPR, electron paramagnetic resonance, ESI-MS, electrospray ionization-MS, ICP-OES, inductively coupled plasma-optical emission spectroscopy, IscS_*Ec*_, cysteine desulfurase from *Escherichia coli*, Lar, lactate racemase, LarE_*Lp*_, LarE from *Lactobacillus plantarum*, LarE_*Tm*_, LarE from *Thermotoga maritima*, LC, liquid chromatography, MS, mass spectrometry, NaAD, nicotinic acid adenine dinucleotide, NPN, nickel-pincer nucleotide, P2CMN, pyridinium-3,5-biscarboxylic acid mononucleotide, P2TMN, pyridinium-3,5-bisthiocarboxylic acid mononucleotide, PCTMN, pyridinium-3-carboxy-5-thiocarboxylic acid mononucleotide, UV-vis, UV-visible

## Abstract

Sulfur-insertion reactions are essential for the biosynthesis of several cellular metabolites, including enzyme cofactors. In *Lactobacillus plantarum*, a sulfur-containing nickel-pincer nucleotide (NPN) cofactor is used as a coenzyme of lactic acid racemase, LarA. During NPN biosynthesis in *L. plantarum*, sulfur is transferred to a nicotinic acid–derived substrate by LarE, which sacrifices the sulfur atom of its single cysteinyl side chain, forming a dehydroalanine residue. Most LarE homologs contain three conserved cysteine residues that are predicted to cluster at the active site; however, the function of this cysteine cluster is unclear. In this study, we characterized LarE from *Thermotoga maritima* (LarE_*Tm*_) and show that it uses these three conserved cysteine residues to bind a [4Fe-4S] cluster that is required for sulfur transfer. Notably, we found LarE_*Tm*_ retains all side chain sulfur atoms, in contrast to LarE_*Lp*_. We also demonstrate that when provided with L-cysteine and cysteine desulfurase from *Escherichia coli* (IscS_*Ec*_), LarE_*Tm*_ functions catalytically with IscS_*Ec*_ transferring sulfane sulfur atoms to LarE_*Tm*_. Native mass spectrometry results are consistent with a model wherein the enzyme coordinates sulfide at the nonligated iron atom of the [4Fe-4S] cluster, forming a [4Fe-5S] species, and transferring the noncore sulfide to the activated substrate. This proposed mechanism is like that of TtuA that catalyzes sulfur transfer during 2-thiouridine synthesis. In conclusion, we found that LarE sulfur insertases associated with NPN biosynthesis function either by sacrificial sulfur transfer from the protein or by transfer of a noncore sulfide bound to a [4Fe-4S] cluster.

The biological interconversion of chemical isomers or enantiomers is of fundamental importance to the metabolism of living organisms and has many applications in biocatalysis, biotechnology, and drug discovery (1). Depending on their substrates, isomerases are divided into four subclasses that act on amino acids and derivatives, α-hydroxyacids and derivatives, carbohydrates and derivatives, and other substances. The isomerization of D/L-lactate is catalyzed by lactate racemase (Lar), an enzyme that, in *Lactobacillus plantarum*, is a combination of the LarA protein and a tethered nickel-pincer nucleotide (NPN) cofactor (2). Homologs of LarA use NPN to catalyze racemization and epimerization reactions of a variety of α-hydroxy acid compounds other than lactate (3).

The biosynthesis of the NPN cofactor in *L. plantarum* requires three proteins that are encoded with LarA in the *lar* operon (Fig. 1) (4, 5). LarB is a carboxylase/hydrolase of nicotinic acid adenine dinucleotide (NaAD), forming pyridinium-3,5-biscarboxylic acid mononucleotide (P2CMN) (6). LarE sequentially converts the two carboxylic acids of P2CMN into thiocarboxylic acids, producing first pyridinium-3-carboxy-5-thiocarboxylic acid mononucleotide (PCTMN) and then pyridinium-3,5-bisthiocarboxylic acid mononucleotide (P2TMN) (7). Finally, LarC installs the nickel atom into P2TMN to generate NPN with the metal bonded in a planar arrangement by one carbon and two sulfur atoms of the coenzyme (8). The organometallic cofactor binds to *L. plantarum* LarA with its nickel coordinating His200 and by a thioamide linkage with Lys184. Analogous histidinyl and lysyl residues are found in other LarA homologs; however, NPN is not covalently attached to all proteins with which it associates.

**Figure 1:**
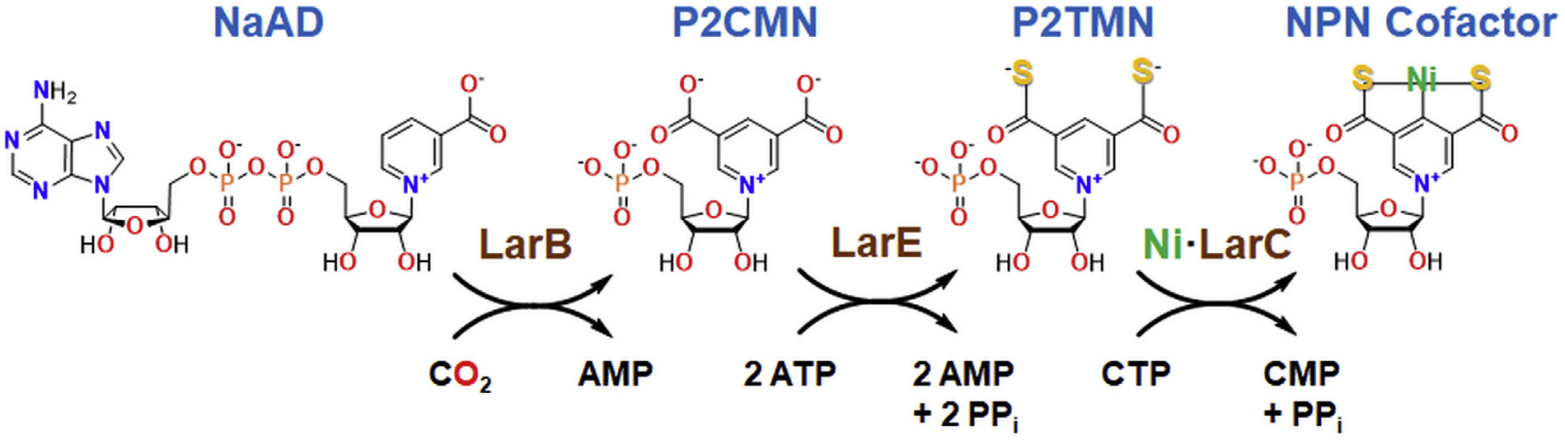
**Biosynthesis of the NPN cofactor.** LarB carboxylates the pyridinium ring and hydrolyzes the phosphoanhydride linkage of nicotinic acid adenine dinucleotide (NaAD) to form pyridinium-3,5-biscarboxylic acid mononucleotide (P2CMN). LarE catalyzes the ATP-dependent conversion of P2CMN into pyridinium-3,5-bisthiocarboxylic acid mononucleotide (P2TMN). LarC inserts nickel into P2TMN to produce the NPN cofactor in a CTP-dependent reaction.

This work focuses on the biosynthetic reaction catalyzed by LarE. Bioinformatics analysis reveals that LarE is a member of the PP-loop pyrophosphatase family that contains a PP-loop SGGXDS motif in its N-terminal region (9). This motif is responsible for catalyzing the ATP-dependent adenylylation of one P2CMN carboxyl group, with the activated intermediate undergoing sulfur transfer to form PCTMN, then a second round of adenylylation and sulfur transfer provides P2TMN. For LarE from *L. plantarum* (LarE_*Lp*_) the sulfur originates from a cysteine residue (Cys176) of the protein, resulting in the formation of a dehydroalanine residue in LarE_*Lp*_, making it a sacrificial sulfur transferase (7) (Fig. 2). LarE_*Lp*_ is structurally related to other ATP-dependent sulfur transferases that contain [4Fe-4S] clusters or that generate persulfides at their active sites, such as TtuA, MnmA, and ThiI (10). Therefore, we speculated that other LarE homologs may bind a [4Fe-4S] cluster or generate a persulfide instead of using an active site cysteine residue for catalytic sulfur transfer.

**Figure 2:**
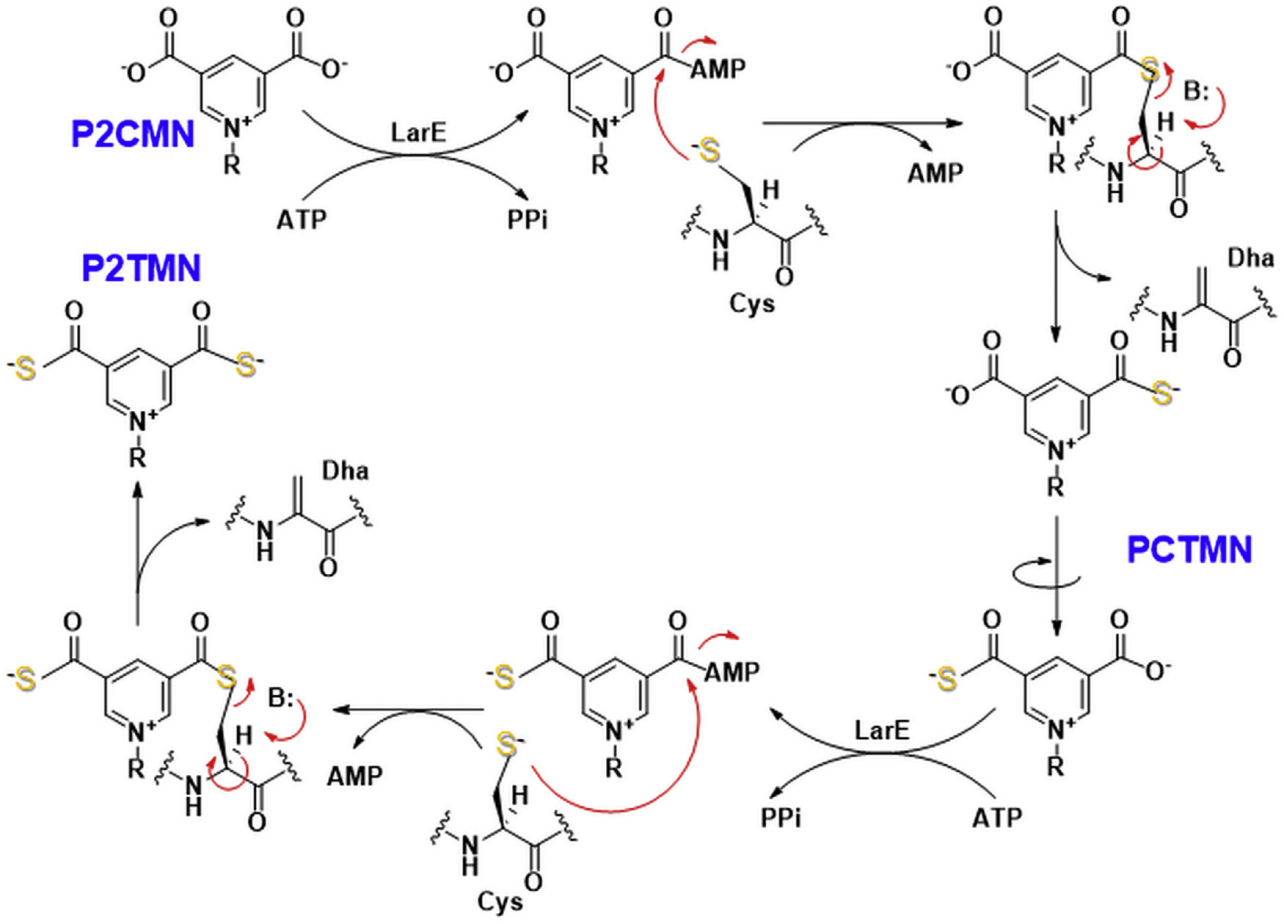
**LarE**_***Lp***_**catalyzed biosynthesis of P2TMN from P2CMN.** One carboxyl group of P2CMN is adenylylated to provide an activated substrate that is attacked by a cysteinyl residue of the enzyme, forming a covalent adduct. Transfer of the enzyme sulfur atom generates protein that contains dehydroalanine (Dha) while producing pyridinium-3-carboxy-5-thiocarboxylic acid mononucleotide (PCTMN). Another subunit of the enzyme then activates PCTMN, forming a covalent adduct. Subsequent sacrifice of the cysteinyl sulfur atom forms P2TMN and a second Dha-containing protein subunit. P2CMN, pyridinium-3,5-biscarboxylic acid mononucleotide; P2TMN, pyridinium-3,5-bisthiocarboxylic acid mononucleotide.

In this study, we identify LarE from *Thermotoga maritima* (LarE_*Tm*_) as a [4Fe-4S] cluster-containing enzyme that coordinates the cluster using three cysteine residues. This conclusion is based on a combination of UV-visible (UV-vis) absorption and electron paramagnetic resonance (EPR) spectroscopies coupled with iron and sulfide analyses as well as site-directed mutagenesis. In addition, we used *in vitro* enzyme assays to demonstrate that the cluster of LarE_*Tm*_ is essential for its sulfur transferase activity. Unlike LarE_*Lp*_, LarE_*Tm*_ does not sacrifice a cysteinyl sulfur atom during catalysis. Rather, we show the [4Fe-4S] cluster is used to catalyze multiple rounds of sulfur transfer when provided with L-cysteine in the presence of cysteine desulfurase from *Escherichia coli* (IscS_*Ec*_). We used mass spectrometry (MS) studies to reveal that IscS_*Ec*_ can directly transfer sulfane sulfur to LarE_*Tm*_. From these results, we speculate that LarE_*Tm*_ uses three conserved cysteines of the enzyme to bind the [4Fe-4S] cluster and coordinates a noncore sulfur atom at the fourth iron site, with the additional sulfur atom used to attack the activated substrate for sulfur transfer. This novel mechanism for LarE_*Tm*_ resembles the sulfur-insertion reactions of thionucleotide-tRNA biosynthetic enzymes that also use tricoordinated [4Fe-4S] clusters to transfer a noncore sulfide (11, 12).

## Results

### Sequence analysis suggests that most LarE homologs possess a tri-Cys-bound iron-sulfur cluster

**Figure 3:**
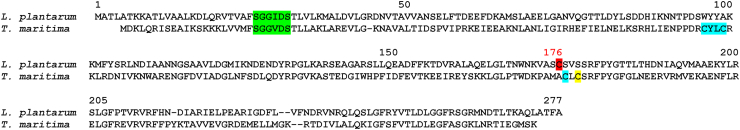
**Sequence comparison of LarE**_***Lp***_**and LarE**_***Tm***_**.** The *green* and *cyan highlights* are the motifs depicted by *green* and *blue* segments in Fig. S1. The sulfur-donating cysteine residue of LarE_*Lp*_ is highlighted in *red*. The fourth cysteine residue adjacent to the CxxC-C motif in LarE_*Tm*_ is highlighted in *yellow*. LarE_*Lp*_, LarE from *Lactobacillus plantarum*; LarE_*Tm*_, LarE from *Thermotoga maritima*.

**Figure 4:**
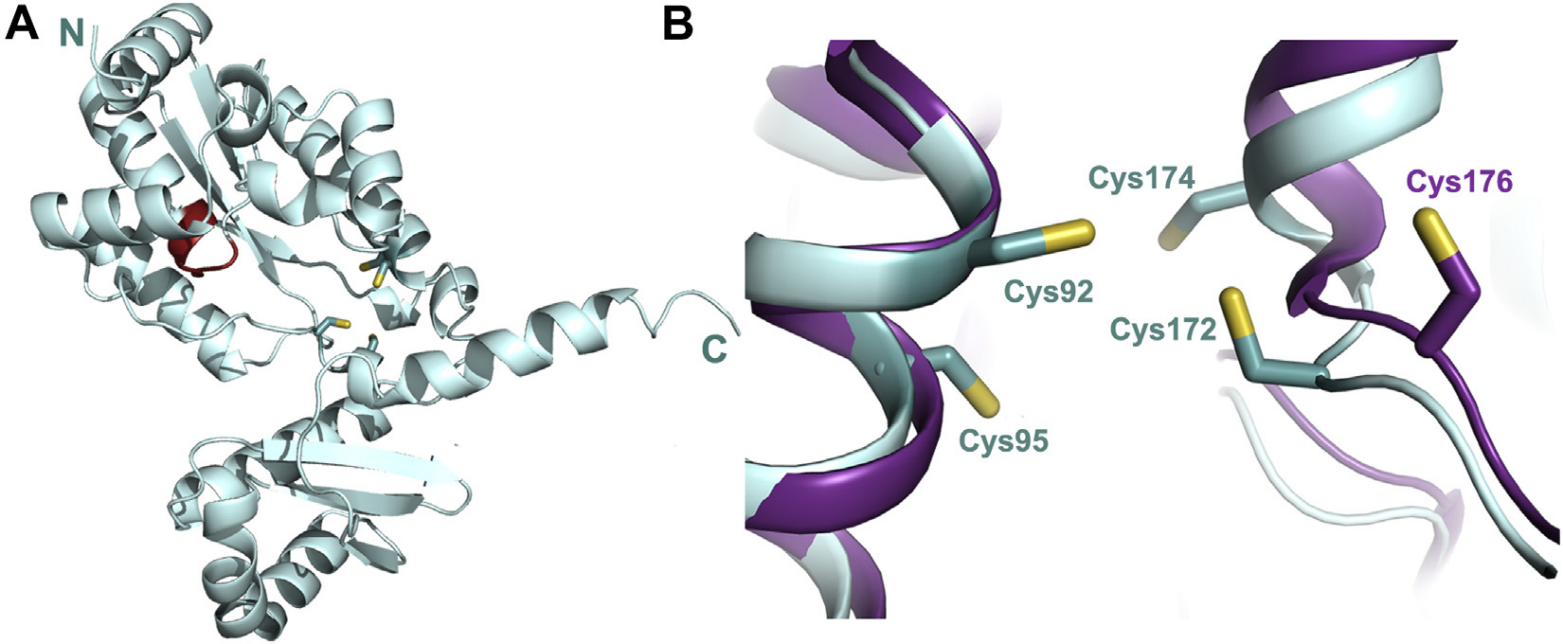
**Homology model of LarE**_***Tm***_**.***A*, cartoon depiction of the LarE_*Tm*_ subunit model with the amino and carboxyl termini indicated (N and C, respectively), the PP-loop shown in *red*, and the four cysteine residues illustrated as *sticks*. *B*, comparison of the 4-cysteine cluster in the LarE_*Tm*_ homology model (*light teal*) *versus* the corresponding region of LarE_*Lp*_ (*purple*) that contains a single cysteine residue. LarE_*Tm*_, LarE from *Thermotoga maritima*.

We analyzed sequences of LarE from selected bacteria and archaea (Fig. S1) and found that most homologs contain two conserved motifs: SGGXDS (P-loop motif, shown beneath the *green rectangular box*) and two cysteines in a CXXC sequence with another cysteine located more distantly (*i.e.*, the CXXC-C motif, shown beneath the *blue rectangular boxes*). In addition, a fourth cysteine residue is located adjacent to the CXXC-C motif in LarE sequences from *T. maritima*, *Ignisphaera aggregans*, and *Deltaproteobacteria bacterium* (shown by the *yellow star*), whereas the other sequences possess Ala, Ser, or Leu at this position. From the structure of LarE_*Lp*_ in complex with Mg·ATP (Protein Data Bank ID: 5UDS), we know the SGGXDS motif (9) is responsible for binding ATP, which is essential for activity (7). The LarE_*Tm*_ and LarE_*Lp*_ sequences both contain the SGGXDS motif, but LarE_*Lp*_ lacks the CXXC-C motif (Fig. 3). Moreover, a single residue shift occurs when comparing the active site Cys176 of LarE_*Lp*_ to the third conserved Cys residue of the CXXC-C motif in LarE_*Tm*_. We used Alphafold2 (13) to build a homology model of LarE_*Tm*_ (Fig. 4*A*) that reveals a clustering of the four cysteine residues (Fig. 4*B*). Ser180, Arg212, and Arg214 of LarE_*Lp*_ are proposed to bind the phosphate of the substrate P2CMN; these residues are conserved in LarE_*Tm*_, so P2CMN is likely to bind in a similar manner. Significantly, the clustering of the three conserved cysteine residues is reminiscent of the situation for other PP-loop pyrophosphatase family members that catalyze sulfur transfer reactions using tricoordinated iron-sulfur clusters (10). Thus, we speculated that LarE homologs containing CXXC-C motifs, including that from *T. maritima*, bind Fe-S clusters, which participate in their sulfur transferase reactions, thus avoiding the need to forfeit the sulfur atom of a thiolate side chain.

### LarE_*Tm*_ does not sacrifice a cysteinyl sulfur atom

His_6_-tagged LarE_*Tm*_ was isolated from *E. coli* and shown by electrospray ionization-MS (ESI-MS) to have a mass of 31,732 Da, consistent with the expected size of 31,739 Da (Fig. S2). The enzyme was reacted with ATP, MgCl_2_, and P2CMN (produced by incubation of LarB with NaAD and bicarbonate), as previously described (8). The His_6_-tagged LarE_*Tm*_ from the reaction mixture was directly examined by ESI-MS or characterized after further purification as described earlier (5). The mass of the protein remained constant throughout the reaction time, indicating that LarE_*Tm*_ does not sacrifice a cysteinyl sulfur atom during the reaction to produce P2TMN (Fig. S2). By contrast, we used the same approach to confirm that LarE_*Lp*_ catalyzed the previously reported sacrificial sulfur transfer reaction (5). The ESI-MS results for His_6_-tagged LarE_*Tm*_ also confirm the homogeneity of the sample. Analysis by size-exclusion chromatography-multiangle light scattering demonstrated that the protein exists as dimer (*M*_r_ = 61,130 ± 36 Da) in solution (Fig. S3).

### LarE_*Tm*_ possesses an oxygen-labile [4Fe-4S] cluster

**Figure 5:**
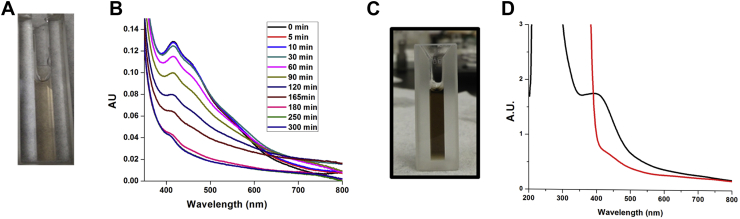
**UV-visible spectroscopy indicates the presence of an oxygen-labile and redox-active [Fe-S] cluster in His**_**6**_**-tagged LarE**_***Tm***_**.***A*, aerobically purified LarE_*Tm*_ (160 μM subunit concentration) was *brownish* in color. *B*, the spectrum of LarE_*Tm*_ decreased in intensity when incubated in air up to 300 min. *C*, aerobically purified LarE_*Tm*_ (200 μM) that had been subjected to cluster assembly conditions yielded a much more intense *brown* spectrum. *D*, the addition of Na_2_S_2_O_4_ (8 mM) to the cluster-assembled LarE_*Tm*_ led to a bleaching of the spectrum (*black* to *red*). All samples were prepared in 100 mM Tris buffer at pH 7.2. LarE_*Tm*_, LarE from *Thermotoga maritima*.

**Figure 6:**
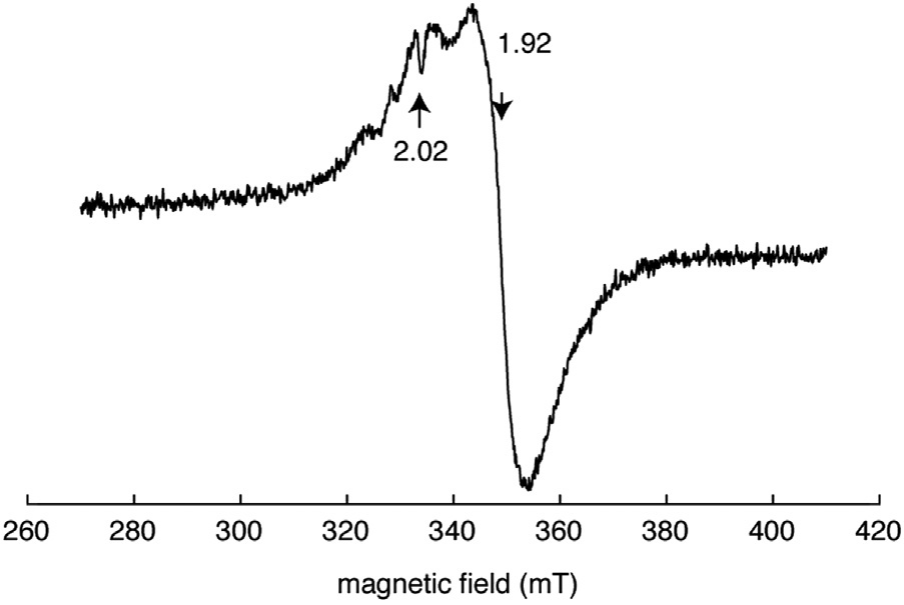
**EPR spectrum of dithionite-reduced, cluster-assembled, His**_**6**_**-tagged LarE**_***Tm***_**.** The spectrum was obtained under the following conditions: microwave frequency, 9.3941 GHz; microwave power, 0.25 mW; magnetic field modulation amplitude, 1.2 mT; sample temperature, 10 K. EPR, electron paramagnetic resonance; LarE_*Tm*_, LarE from *Thermotoga maritima*.

The aerobically purified protein (subunit concentration of 160 μM) was brownish in color (Fig. 5*A*), but the intensity of the broad absorption (maximum at ∼410 nm) decreased with time when exposed to air (Fig. 5*B*). When aerobically purified His_6_-tagged LarE_*Tm*_ (200 μM) was anaerobically incubated with iron and L-cysteine in the presence of IscS_*Ec*_ (*i.e.*, cluster assembly conditions), the brownish color and the broad shoulder at 410 nm were greatly increased in intensity (Fig. 5*C*), consistent with an enhanced amount of cluster compared to the starting sample with its partially occupied metallocenter. The spectrum of anaerobic cluster-assembled LarE_*Tm*_ (Fig. 5*D*) differed from that of the aerobically purified protein (Fig. 5*B*), presumably because the aerobic sample possessed some damaged cluster (*e.g.*, [3Fe-4S], [2Fe-2S], and perhaps other species). Anaerobic addition of sodium dithionite into cluster-assembled LarE_*Tm*_ (400 μM) bleached the protein color and greatly diminished the UV-vis absorption (Fig. 5*D*). Inductively coupled plasma-optical emission spectroscopy (ICP-OES) revealed 4.1 ± 0.2 Fe atoms per subunit, and chemical analysis indicated the presence of 3.9 ± 0.2 inorganic sulfur atoms per monomer for protein that was analyzed after *in vitro* cluster assembly. These values are consistent with the presence of a [4Fe-4S] cluster in each subunit. We investigated the oxidation state properties of the [4Fe-4S] cluster in cluster-assembled LarE_*Tm*_ by EPR spectroscopy. The initial spectrum displayed low intensity signals near *g* ∼2 at 10 K that are consistent with a small amount of the [4Fe-4S] cluster having been damaged and oxidized to form a [3Fe–4S]^1+^ cluster and radicals (14). After treatment of the LarE_*Tm*_ sample with low concentrations of sodium dithionite, we obtained an EPR spectrum (Fig. 6) with *g*_‖_ = 2.02 and *g*_⊥_ = 1.92, consistent with a [4Fe-4S]^+1^ cluster (15), along with sharp radical signal (*g* = 2.00) that we attribute to unreacted SO_2_^-⋅^ or a secondary radical (16, 17).

### The [4Fe-4S] cluster of LarE_*Tm*_ is required for its sulfur transferase activity

**Figure 7:**
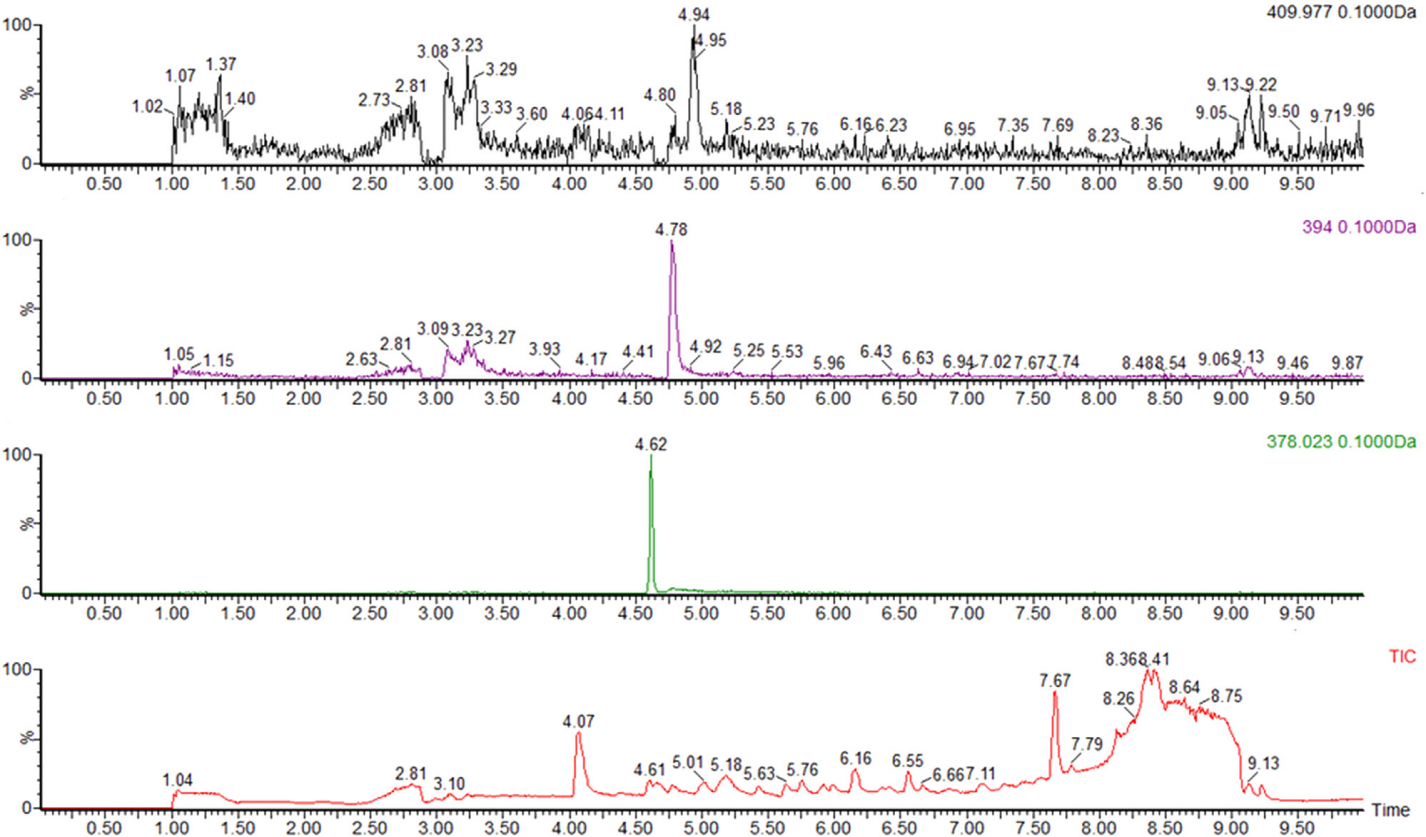
**His**_**6**_**-tagged LarE**_***Tm***_**converts P2CMN into PCTMN and P2TMN as shown by LC-ESI-MS chromatography.** Selected ion monitoring of P2TMN (*top* scan at 410.0 Da), PCTMN (second scan at 394.0 Da), and P2CMN (third scan at 378.0 Da) elute at 4.94, 4.78, and 4.62 min, respectively. The *bottom panel* shows the total ion chromatogram (TIC). The reaction was performed using freshly purified LarE_*Tm*_ (∼1 mM, isolated aerobically) in 100 mM Tris, pH 7.2 buffer containing 20 mM MgCl_2_ and 2 mM ATP. ESI-MS, electrospray ionization-mass spectrometry; LarE_*Tm*_, LarE from *Thermotoga maritima*; LC, liquid chromatography; P2CMN, pyridinium-3,5-biscarboxylic acid mononucleotide; P2TMN, pyridinium-3,5-bisthiocarboxylic acid mononucleotide; PCTMN, pyridinium-3-carboxy-5-thiocarboxylic acid mononucleotide.

**Figure 8:**
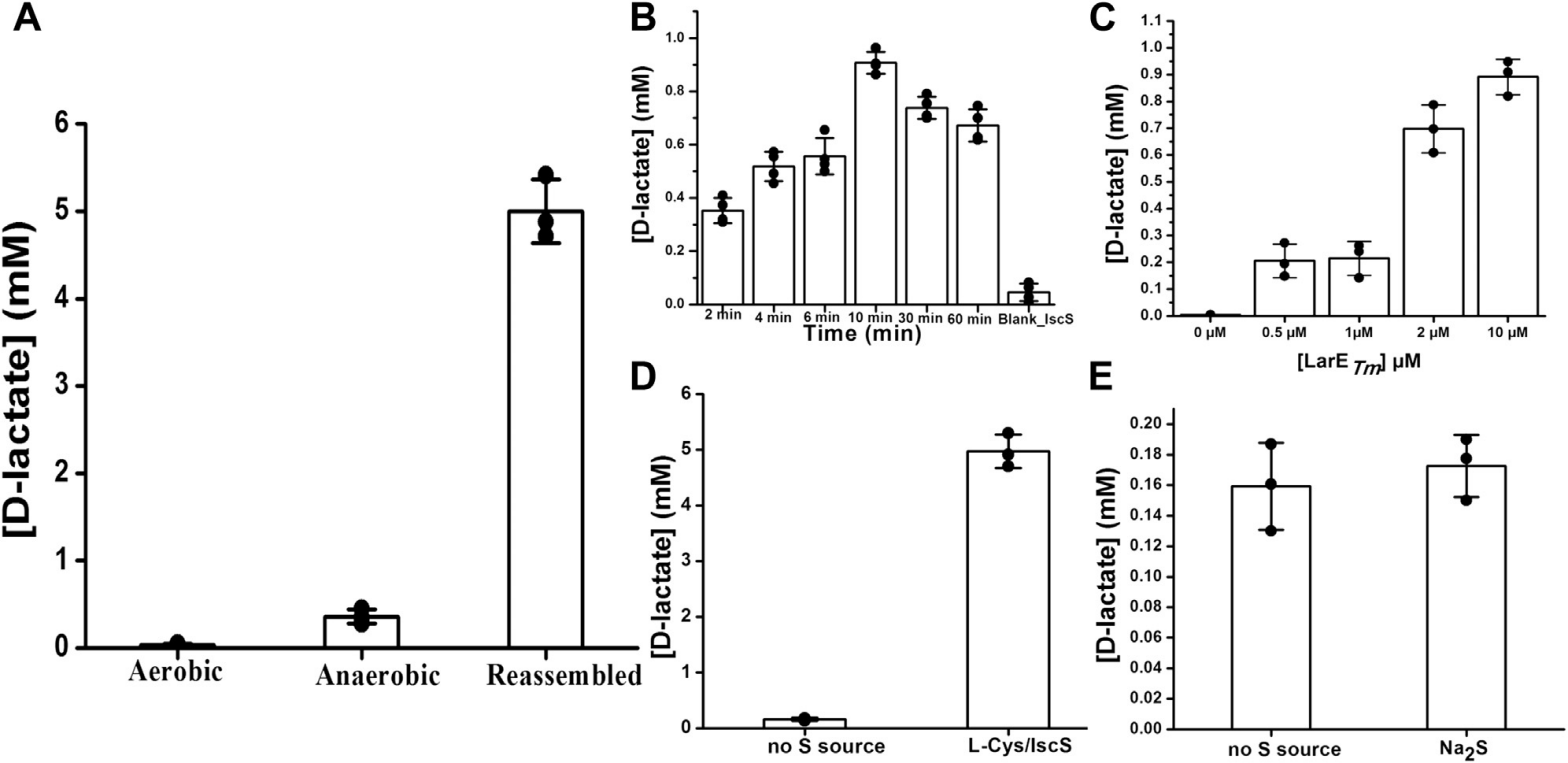
**Sulfur transferase activity of His**_**6**_**-tagged LarE**_***Tm***_**.** LarE_*Tm*_ samples were incubated at room temperature for the indicated time periods with P2CMN (prepared by LarB_*Lp*_-catalyzed transformation of NaAD), 20 mM MgCl_2_, and 2 mM Mg·ATP, then denatured by heat treatment at 80 °C for 10 min. The activities of the LarE_*Tm*_ samples were assessed by an indirect assay in which the LarE_*Tm*_ product, P2TMN, was converted into NPN using LarC_*Lp*_, the cofactor was incorporated into LarA_*Tt*_ apoprotein, and Lar activity was monitored based on the production of D-lactate from 45 mM L-lactate in the starting solution. *A*, comparison of activities after 60 min incubation for three LarE_*Tm*_ samples at 1 mM concentration: prepared aerobically, isolated anaerobically, and purified then subjected to anaerobic cluster-assembly conditions. *B*, time dependence of activity for cluster-assembled LarE_*Tm*_ based on the subsequent conversion of P2TMN to NPN and activation of lactate racemase, monitoring D-lactate production. A low concentration of enzyme (10 μM) was used to allow for kinetic analysis. *C*, effect of the LarE_*Tm*_ enzyme concentration on activity during 10 min of incubation as measured by the downstream Lar activity determination. *D*, effect of added L-Cys (10 mM) plus IscS_*Ec*_ (20 μM) on the activity of LarE_*Tm*_ (1 mM) compared to the activity of enzyme with no sulfur supplementation during a 10 min incubation, as monitored by the resulting Lar activity. *E*, effect of added Na_2_S (10 mM) on the activity of LarE_*Tm*_ (1 mM) compared to the activity of enzyme with no sulfur supplementation during a 10 min incubation, based on the observed Lar activity. The plots include individual data points, the means, and the SDs. LarE_*Tm*_, LarE from *Thermotoga maritima*; NaAD, nicotinic acid adenine dinucleotide; P2CMN, pyridinium-3,5-biscarboxylic acid mononucleotide; P2TMN, pyridinium-3,5-bisthiocarboxylic acid mononucleotide.

In the presence of Mg·ATP, freshly purified LarE_*Tm*_ transformed P2CMN into PCTMN and P2TMN, as detected by selected ion monitoring during liquid chromatography (LC)-ESI-MS (Fig. 7). This enzyme activity was lost after oxygen exposure. The activities of LarE_*Tm*_ samples also were assessed by using an indirect assay that involved the conversion of NaAD to P2CMN by LarB, LarE-catalyzed anaerobic transformation of this substrate to P2TMN, synthesis of NPN from P2TMN by LarC, incorporation of the cofactor into LarA apoprotein from *Thermoanaerobacterium thermosaccharolyticum* (LarA_*Tt*_), and measurement of the resulting Lar activity (Fig. 8*A*). Anaerobic cluster-assembled LarE_*Tm*_ exhibited the greatest activity, whereas significantly reduced levels were observed for anaerobically purified protein, and essentially no activity was detected by this assay in the aerobically purified sample. To further characterize the reaction, we examined different time intervals (Fig. 8*B*) as well as different concentrations of the cluster-assembled LarE_*Tm*_ (Fig. 8*C*). We observed increasing levels of activity for the first 10 min of the reaction, but longer time periods led to a small reduction of activity. As expected, using larger amounts of LarE_*Tm*_ led to greater levels of Lar activity; however, a linear dependence was not observed. As a means to potentially allow catalytic, rather than stoichiometric, activity of LarE_*Tm*_, we provided exogenous sulfur sources. Treating LarE_*Tm*_ with L-cysteine and His_6_-tagged IscS_*Ec*_ provided a substantial increase in the levels of Lar activity (Fig. 8*D*). In contrast, provision of Na_2_S as an external sulfur source along with FeCl_3_ did not increase the level of Lar activity over the control (Fig. 8*E*). These results suggest that LarE_*Tm*_ can use L-cysteine as sulfur donor when provided along with His_6_-tagged IscS_*Ec*_, whereas Na_2_S does not serve as the sulfur source for the sulfur transferase activity of this enzyme.

### Mutational analysis of the cysteine codons of LarE_*Tm*_

**Figure 9:**
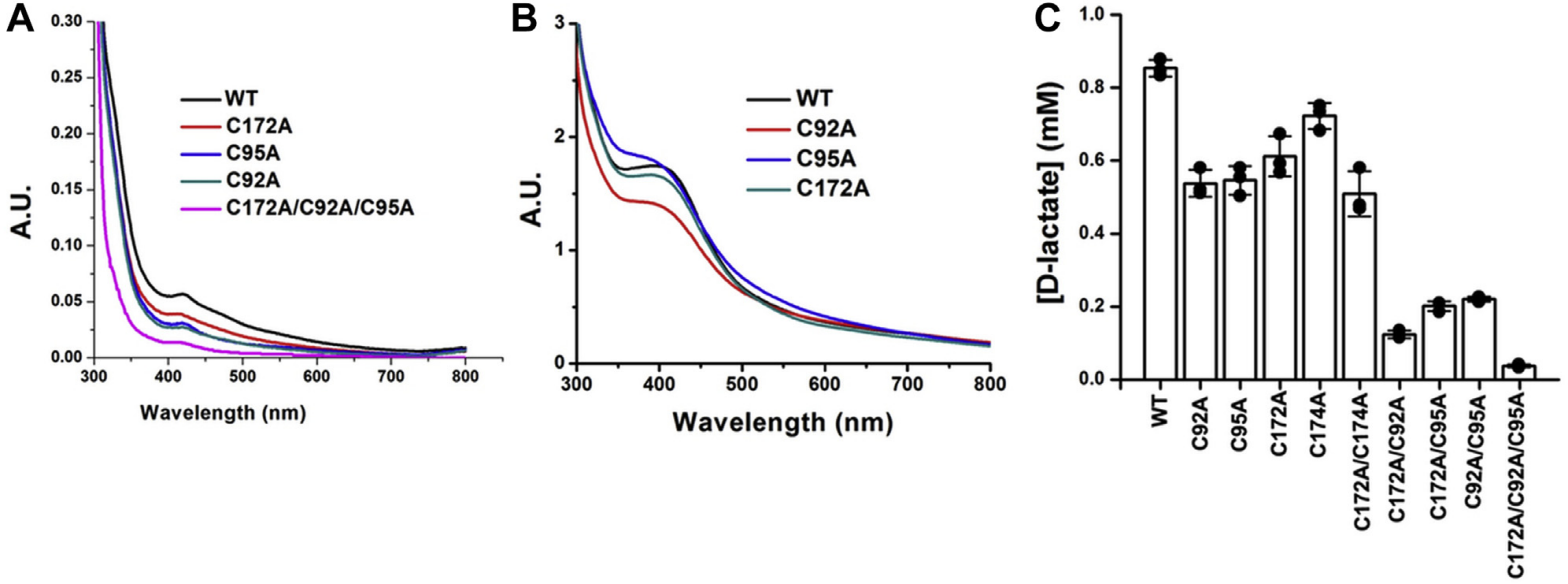
**Mutational analysis of conserved cysteine codons of LarE**_***Tm***_**.***A*, UV-visible spectra were acquired for the control His_6_-tagged LarE (WT) and its C92A, C95A, C172A, and C172A/C92A/C95A variants (100 μM) immediately after purification. *B*, WT, C92A, C95A, and C172A variants were subjected to cluster-assembly conditions and the spectra acquired. *C*, WT LarE and combinations of cysteine variants (all at 7.5 μM) were incubated with P2CMN, 20 mM MgCl_2_, and 2 mM Mg⋅ATP for 10 min and then examined for enzyme activity using the indirect Lar activation assay that monitors D-lactate production. This plot includes individual data points, the means, and the SDs. LarE_*Tm*_, LarE from *Thermotoga maritima*.

To further test the importance of the [4Fe-4S] cluster to the sulfur transferase activity of LarE_*Tm*_, we mutated the gene to substitute each of its three conserved cysteine residues (Cys92, Cys95, and Cys172) with alanine. The three variant proteins were isolated aerobically, UV-vis absorption spectra were acquired (Fig. 9*A*), the samples were subjected to cluster assembly conditions, the modified spectra were acquired (Fig. 9*B*), and the variants were measured for enzymatic activity (Fig. 9*C*). The broad absorption peak at ∼410 nm was decreased, but not eliminated, for each of the variants as initially purified in comparison to the control protein at the same concentration (100 μM). On the basis of the A_410_/A_280_ ratios of the C92A, C95A, and C172A variant proteins and comparison to the WT enzyme, the relative amounts of [4Fe-4S] cluster present were approximately 48%, 54%, and 61%, respectively (Fig. 9*A*). After treatment of the proteins with cluster-assembly conditions, the samples were much more closely matched in their 410 nm absorption with slightly less in the C92A variant (Fig. 9*B*). The enzymatic activities of the cluster-assembled C92A, C95A, and C172A variants (assayed at 7.5 μM concentrations) were 63%, 64%, and 71% of that for the WT enzyme using the Lar assay, respectively (Fig. 9*C*). These activity experiments were performed at least twice and were also reproduced at different assay time periods. The results are consistent with the Cys92, Cys95, and Cys172 residues being ligands of the [4Fe-4S] cluster but indicate that cluster formation and substantial activity is retained even when one of these ligands is removed. The sequence alignment and the LarE_*Tm*_ homology model show the presence of a fourth cysteine residue (Cys174) adjacent to the tri-Cys cluster. To examine whether Cys174 participates in enzymatic activity and whether it might substitute for Cys172 when that residue is altered, we created constructs that encode the C174A variant and the C172A/C174A double variant. As expected, C174A LarE_*Tm*_ retained nearly all (84%) of the control protein activity, whereas the C172A/C174A protein possessed essentially the same Lar activity (59%) as the C172A single variant (Fig. 9*C*). We conclude that Cys174 is not important for LarE_*Tm*_ activity. To further examine the role of the conserved cysteine residues (Cys92, Cys95, and Cys172) we generated all possible combinations of double variants as well as the triple variant. Greatly diminished levels of activity were observed for the double variants (Fig. 9*C*). For the C92A/C95A/C172A variant, the broad absorption peak at ∼410 nm (Fig. 9*A*) and the Lar activity (Fig. 9*C*) were nearly eliminated, confirming the critical role of these residues in LarE_*Tm*_ activity.

### Cysteine desulfurase can supply sulfur to LarE_*Tm*_

**Figure 10:**
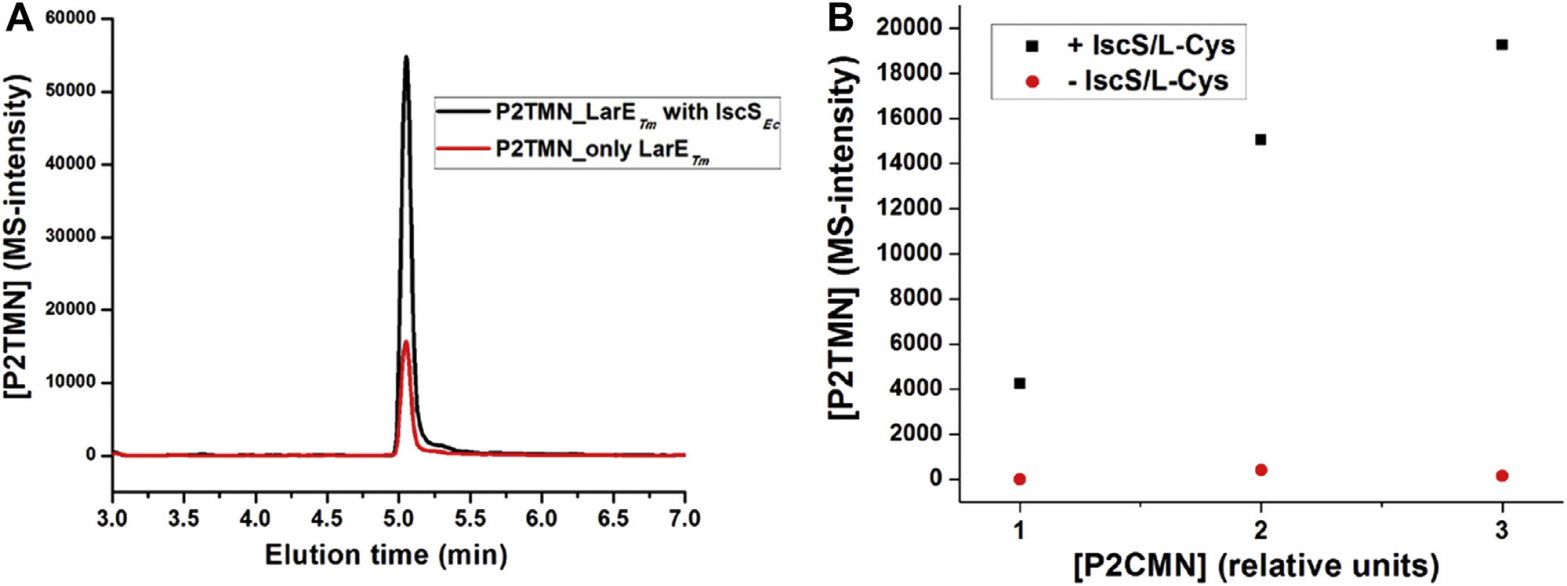
**The relative amount of P2TMN produced by LarE**_***Tm***_**increases in the presence of IscS**_***Ec***_**and L-cysteine.***A*, the LC-MS intensity values of the P2TMN product were compared for His_*6*_-tagged LarE_*Tm*_ (10 μM) incubated with P2CMN, 2 mM ATP, and 20 mM MgCl_2_ for 120 min (*red line*) *versus* an identical sample incubated with the same conditions for 60 min, followed by 60 min incubation with added His_6_-tagged IscS_*Ec*_ (100 μM) and L-cysteine (10 mM) (*black line*). *B*, similar P2TMN LC-MS intensity analyses were carried out for samples containing or lacking IscS_*Ec*_ plus L-cysteine using three concentrations of substrate P2CMN (shown as relative units because a standard is not available for comparison) for a total incubation period of 120 min. IscS_*Ec*_, cysteine desulfurase from *Escherichia coli*; LarE_*Tm*_, LarE from *Thermotoga maritima*; P2CMN, pyridinium-3,5-biscarboxylic acid mononucleotide; P2TMN, pyridinium-3,5-bisthiocarboxylic acid mononucleotide.

As indicated previously, the inclusion of His_6_-tagged IscS_*Ec*_ and L-cysteine greatly enhanced the activity of His_6_-tagged LarE_*Tm*_ when assessed using the indirect Lar activation assay. This finding led us to consider whether IscS_*Ec*_ may donate sulfur to LarE_*Tm*_ for subsequent incorporation into the cofactor. Genes encoding two cysteine desulfurases (*tmari_1700* and *tmari_1378*) are located distant from that encoding LarE_*Tm*_ (*tmari_0968*) in the *T. maritima* genome, with the product from *tmari_1700* (IscS_*Tm*_) sharing 40% sequence identity with IscS_*Ec*_ (155 matches out of 384 residues) (Fig. S4*A*). The crystal structure of IscS_*Ec*_ (Protein Data Bank ID: 3LVK) (Fig. S4*B*) was used to create a homology model of IscS_*Tm*_ (prepared by SWISS MODEL) (18) (Fig. S4*C*) that depicts the conserved position of a cysteine residue, which would accept sulfane sulfur from cysteine during the pyridoxal phosphate-dependent reaction (19). We used small molecule LC-MS analysis to explore whether IscS_*Ec*_ can directly supply sulfur to LarE_*Tm*_ for subsequent biosynthesis of the NPN cofactor in a manner similar to its reported roles during biosynthesis of other sulfur-containing cofactors such as iron-sulfur clusters, thiamin, and biotin (20, 21, 22). We incubated the cluster-assembled His_*6*_-tagged LarE_*Tm*_ (10 μM) with P2CMN, 20 mM MgCl_2_, and 2 mM ATP for 60 min inside the anaerobic chamber at room temperature (RT), then added anoxic IscS_*Ec*_ (100 μM) and L-cysteine (10 mM) for another 60 min. Equal volumes of buffer were added to a control sample (lacking L-cysteine and IscS_*Ec*_) that was incubated for a total of 120 min. Analysis of the metabolites by LC-MS revealed a fourfold greater amount of P2TMN produced in the presence of IscS_*Ec*_ and L-cysteine (Fig. 10*A*). The results are shown as relative changes because no standards are available for P2CMN or P2TMN so the concentrations of substrate provided and product formed are not established. To confirm and extend this result, we varied the relative amount of P2CMN by adding one, two, and three equivalent volumes to the reactions during the 60 min incubation, while maintaining a constant concentration of LarE_*Tm*_ (10 μM). The presence of IscS_*Ec*_ and L-cysteine led to clear increases in P2TMN production as more P2CMN substrate was provided, whereas in their absence, the production of P2TMN was very low at all levels of P2CMN provided (Fig. 10*B*).

### Direct donation of sulfane sulfur from the IscS_*Ec*_ persulfide

**Figure 11:**
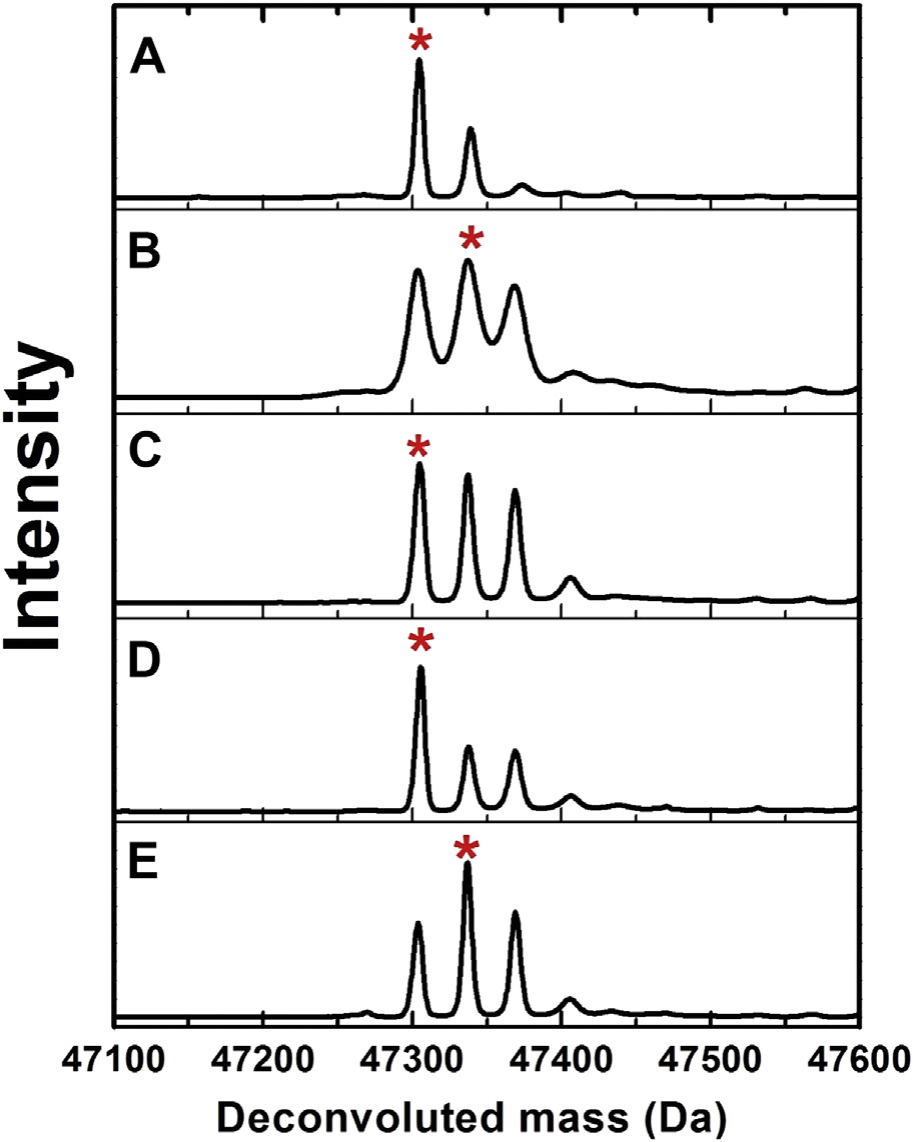
**Sulfane sulfur transfer from persulfidated IscS**_***Ec***_**to LarE.** ESI-MS data were collected and the results deconvoluted for (*A*) as purified His_6_-tagged IscS_*Ec*_, (*B*) the purified IscS_*Ec*_ treated with L-cysteine to produce the IscS with one or more added sulfane sulfur atom (IscS-S), (*C*) IscS-S incubated with aerobically-purified His_6_-tagged LarE_*Tm*_, (*D*) IscS-S incubated with anaerobically purified and cluster-assembled His_6_-tagged LarE_*Tm*_, and (*E*) IscS-S incubated with bovine serum albumin. The concentrations of IscS_*Ec*_, LarE_*Tm*_, and bovine serum albumin were each 10 μM and that of L-cysteine was 0.5 mM. Major peaks have been marked with *red stars*. ESI-MS, electrospray ionization-mass spectrometry; IscS_*Ec*_, cysteine desulfurase from *Escherichia coli*; LarE_*Tm*_, LarE from *Thermotoga maritima*.

The increase in the relative amount of P2TMN produced in the presence of IscS_*Ec*_ and L-cysteine is consistent with a recycling of sulfur-depleted LarE_*Tm*_ by sulfur transfer from an IscS_*Ec*_ persulfide. We used an ESI-MS approach (23) to examine the mass changes of His_6_-tagged IscS_*Ec*_ during the postulated transfer of sulfane sulfur to LarE_*Tm*_. The deconvoluted mass spectrum of as-purified His_6_-tagged IscS_*Ec*_ was comprised predominantly of the monomer molecular ion peak at 47,304 Da, along with a small peak at 47,339 Da and a slight feature at 47,374 Da (Fig. 11*A*). This spectrum is in excellent agreement with the theoretical mass (47,302.94 Da) for the IscS_*Ec*_ sequence, which includes a linker to the His_6_ tag, along with small amounts of sulfane sulfur bound to the protein as purified. After incubating the IscS_*Ec*_ with excess L-cysteine for 60 min at RT and removing excess L-cysteine with a PD-10 desalting column, a large fraction of the sample had shifted in mass to molecular ion peaks at 47,336 Da and 47,369 Da (Fig. 11*B*). These changes represent increases of 32 and 65 Da and are consistent with additions of one and two sulfane sulfur atoms to the protein, forming the persulfide (IscS-S) and a larger species. The addition of a stoichiometric amount of aerobically purified His_6_-tagged LarE_*Tm*_ to this IscS-S sample led to a small shift in the proportions of the three species, increasing the relative amount of free IscS_*Ec*_ (Fig. 11*C*). This shift was much more pronounced when adding a stoichiometric amount of anaerobically purified and cluster-assembled LarE_*Tm*_ that possessed greater levels of intact [4Fe-4S] cluster (Fig. 11*D*). In contrast to these changes, the addition of a stoichiometric amount of bovine serum albumin had no effect on the molecular ion peaks in the sample containing IscS-S (Fig. 11*E*). These results suggest that persulfidated IscS_*Ec*_ can catalyze direct transfer of sulfane sulfur to LarE_*Tm*_. As the shift for IscS_*Ec*_ is more pronounced upon incubation with cluster-assembled LarE_*Tm*_ (Fig. 11*D*), it is reasonable to propose that sulfane sulfur has been transferred to the cluster.

### Formation of a [4Fe-5S] cluster in LarE_*Tm*_

**Figure 12:**
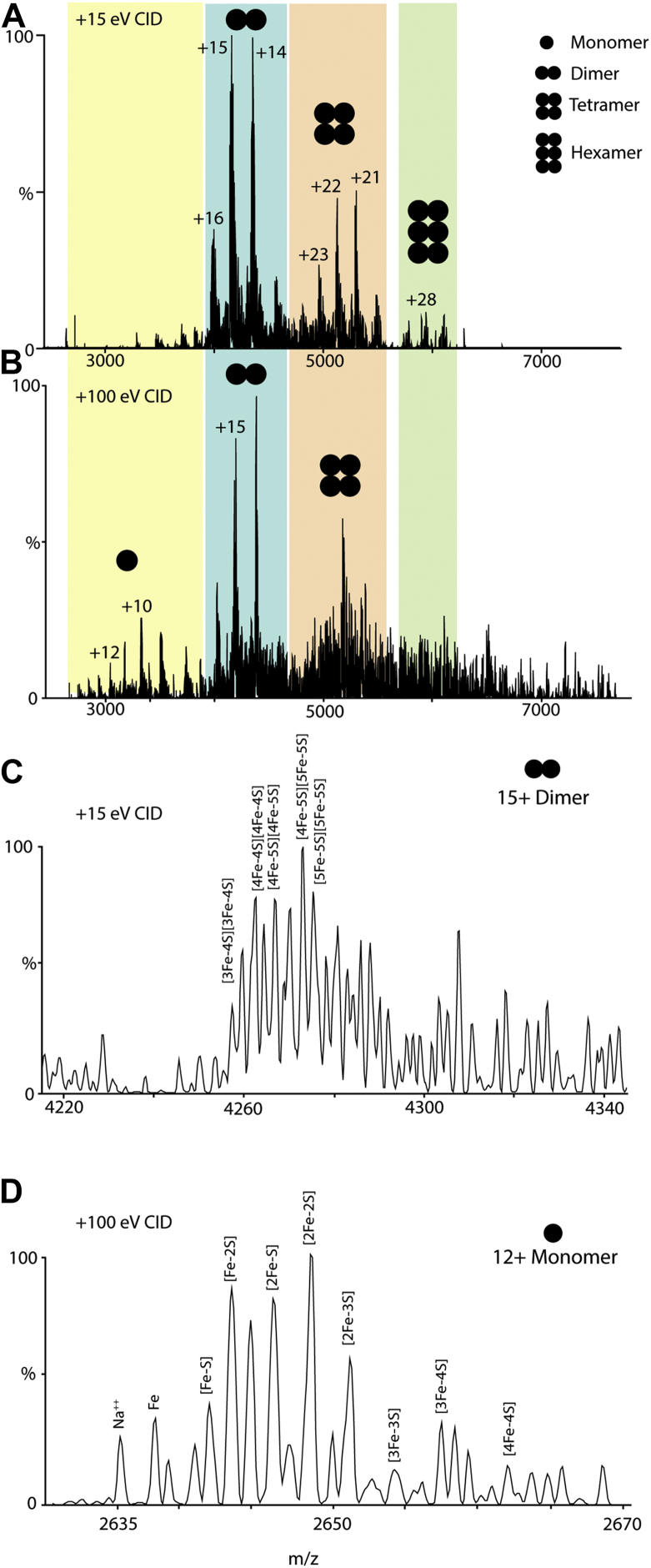
**Native mass spectrometry of LarE**_***Tm***_**.** The figure illustrates the abundance of LarE_*Tm*_ with bound iron and sulfur atoms. *A*, when using a CID of 15 eV, the dimer, tetramer, and hexamer oligomeric states are observed, but the spectrum is void of monomeric signals. *B*, using a CID of 100 eV, the monomeric species becomes evident. *C*, focusing on the 15+ charge state of the dimeric species, multiple Fe-S stoichiometries are observed. *D*, at the *high* CID energy of 100 eV, Fe-S stoichiometries are recorded for the 12+ charge state of monomeric LarE_*Tm*_. LarE_*Tm*_, LarE from *Thermotoga maritima*; CID, collision-induced dissociation.

The lability of the [4Fe-4S] cluster in His_6_-tagged LarE_*Tm*_ precluded use of the aforementioned denaturing ESI-MS method to assess whether sulfane sulfur adds to the *T. maritima* protein. Rather, we used nondenaturing nano–ESI-MS of cluster-assembled His_6_-tagged LarE_*Tm*_ after anaerobic incubation with IscS_*Ec*_ and L-cysteine (Fig. 12). The native MS data reveal signals associated with dimer, tetramer, and hexameric forms of LarE when using 15 eV for collision-induced dissociation (CID). When subjected to a CID energy of 100 eV, signals appear for what is assigned to the LarE monomer, as is typical for such data acquired for noncovalent protein complexes (24). By focusing our analysis on signals for the 15+ charge state of LarE dimers and the 12+ charge state of LarE monomers, we obtained the iron and sulfur stoichiometries associated with the subunits. For this analysis, the numbers of iron and sulfur atoms indicated within the brackets represent the numbers of these atoms bound per subunit and do not necessarily equate to the type of bound iron-sulfur cluster. The observed stoichiometries ranged from [Fe-S] to [4Fe-4S] for the LarE monomer and from [3Fe-4S][3Fe-4S] to [5Fe-5S][5Fe-5S] for LarE dimers (Fig. 12 and Tables S1 and S2). It is possible that the high CID energies required to initiate monomer ejection from the complexes interrogated here resulted in some loss of iron and sulfur from the LarE monomers, giving rise to the lower stoichiometries in these species when compared to the LarE dimer. In addition, it is plausible that iron released from damaged clusters binds adventitiously to some subunits, accounting for subunits associated with five metal ions.

The native MS data revealed prominent peaks that are increased in mass by 384 Da with respect to the apoprotein. A [4Fe-4S] cluster possesses a mass of 352 Da (25), which is also detected, so the 384 Da mass shift is suggestive of a sulfide bound to such a cluster forming a [4Fe-5S] species. We propose that the dimer species labeled [4Fe-4S][4Fe-4S] and [4Fe-5S][4Fe-5S] contain these types of clusters in the two subunits. Additionally, we suggest that dimer species labeled [4Fe-5S][5Fe-5S] and [5Fe-5S][5Fe-5S] each contain two copies of the [4Fe-5S] cluster with adventitious iron atoms bound to one or two of the subunits. We recognize that as an alternative to a [4Fe-5S] cluster, subunits containing a [4Fe-4S] cluster and a cysteine persulfide cannot be ruled out (25).

## Discussion

We have provided evidence that the sulfur transferase activity of LarE_*Tm*_ requires the presence of a [4Fe-4S] cluster that is tricoordinated by three cysteine residues. The cluster is oxygen labile and the protein cluster-binding site is only fully occupied when LarE_*Tm*_ is subjected to cluster-assembly conditions. The labile nature of the cluster results in the EPR spectroscopic detection of small amounts of oxidized [3Fe–4S]^1+^ cluster in the anaerobically purified enzyme, conditions when most of the protein contains diamagnetic [4Fe-4S]^2+^ metallocenter, whereas a more intense spectrum is generated in the reduced sample due to a [4Fe-4S]^1+^ state. Of interest, the presence of a [4Fe-4S] cluster in LarE_*Tm*_ resembles the situation for TtuA, a sulfur transferase involved in 2-thiouridine biosynthesis that binds its cluster using three cysteine residues (26, 27, 28).

Our analysis of LarE_*Tm*_ variants confirms the requirement of Cys92, Cys96, and Cys172, but not Cys174, for coordinating the [4Fe-4S] cluster and conferring activity. Individual substitutions of the cluster ligands reduce the amount of cluster in aerobically purified enzyme samples, but when subjected to cluster assembly conditions, the variant proteins exhibit nearly full incorporation of the cluster according to the intensity of the UV-vis spectra. Furthermore, the activity of these single cysteine variant proteins was only modestly reduced compared to the native protein. Double cysteine variants of LarE_*Tm*_ are more greatly affected and only the triple cysteine variant fails to acquire a cluster or exhibit activity. Somewhat analogous results have been observed for other proteins; for example, variants of the HBx protein of hepatitis B virus where single substitution of cluster ligands did not affect cluster incorporation, and complete abolishment of cluster binding required substitution of all ligands (29).

In the presence of IscS_*Ec*_ and L-cysteine, the activity of LarE_*Tm*_ dramatically improves, consistent with donation of sulfane sulfur from IscS_*Ec*_ to LarE_*Tm*_. Indeed, we used ESI-MS analyses to demonstrate that the persulfide sulfur atom of IscS_*Ec*_ is significantly lost only when incubated with the [4Fe-4S]-containing form of LarE_*Tm*_ but not with the cluster-free protein or with the control protein bovine serum albumin. Furthermore, analysis of the resulting sample by anaerobic, nano–ESI-MS provided evidence consistent with the formation of LarE_*Tm*_ possessing a [4Fe-5S] cluster, although a [4Fe-4S] cluster plus a persulfide cannot be ruled out. Surprisingly, the native LarE_*Tm*_ MS study revealed peaks for the dimer, tetramer, and hexamer species. The crystal structure of LarE_*Lp*_ reveals a hexamer that forms by head-to-head interaction of two trimers (7). The lack of a trimeric species for LarE_*Tm*_ may indicate that it has a distinct quaternary structure.

**Figure 13:**
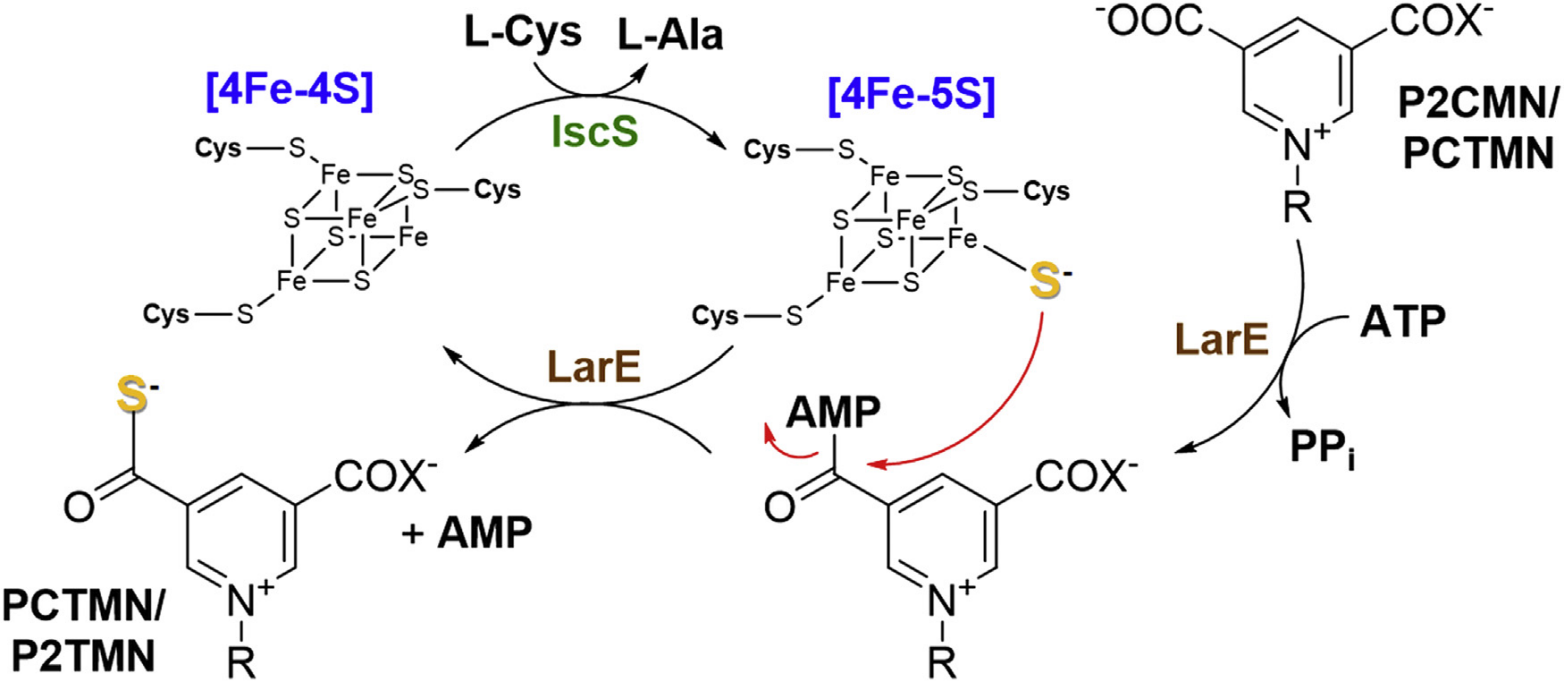
**Proposed mechanism of LarE**_***Tm***_**.** We hypothesize that LarE_*Tm*_ activates P2CMN or PCTMN by adenylylation (*right portion* of the scheme), in the same manner as demonstrated for the LarE_*Lp*_ protein. Rather than sacrificing a cysteinyl sulfur atom, however, LarE_*Tm*_ attacks the activated substrate using a noncore sulfide bound to the open iron site in a tricoordinated [4Fe-4S] cluster, that is, a [4Fe-5S] cluster. The resulting [4Fe-4S]-containing enzyme species lacking the noncore sulfide is restored to the active species by acquiring a sulfane sulfur atom provided from an IscS persulfide that transiently forms as it catalyzes its cysteine desulfurase activity. Two rounds of this mechanism are required to convert P2CMN to P2TMN. LarE_*Tm*_, LarE from *Thermotoga maritima*; P2CMN, pyridinium-3,5-biscarboxylic acid mononucleotide; P2TMN, pyridinium-3,5-bisthiocarboxylic acid mononucleotide; PCTMN, pyridinium-3-carboxy-5-thiocarboxylic acid mononucleotide.

We propose that a persulfide sulfur atom on IscS_*Ec*_ transfers to the open iron coordination site on the [4Fe-4S] cluster of LarE_*Tm*_ as a noncore sulfide atom (Fig. 13). Formation of this [4Fe-5S] cluster would presumably require additional reductant, available from the excess L-cysteine in the activation mixture. We further propose that the cluster-coordinating sulfide attacks the adenylylated P2CMN, formed *via* the same reaction as that catalyzed by LarE_*Lp*_, to produce PCTMN and AMP. A second round of sulfur transfer from the IscS_*Ec*_ persulfide to the [4Fe-4S] cluster of LarE_*Tm*_ regenerates the [4Fe-5S] cluster that reacts with activated PCTMN to form P2TMN. Significantly, this mechanism avoids the conversion of a cysteinyl residue into a dehydroalanine side chain as seen in LarE_*Lp*_. We verified by ESI-MS the retention of all cysteinyl sulfur in LarE_*Tm*_ and its loss from LarE_*Lp*_. It is remarkable that the same overall protein architecture is used for two such distinct mechanisms for transferring sulfur into P2CMN to make P2TMN.

Our demonstration of two distinct LarE sulfur-transfer mechanisms for the biosynthesis of the NPN cofactor has a clear parallel in the two reactions used for sulfur insertion during tRNA thionucleotide synthesis (11, 12). One thiobase-forming mechanism is exemplified by 4-thiouridine synthesis involving the initial activation of the precursor nucleotide using an adenylyltransferase followed by action of a sulfur transferase that catalyzes persulfide attack on the intermediate with release of AMP (30, 31, 32). A second thionucleotide-forming mechanism is associated with [Fe-S] cluster-containing enzymes that resemble LarE_*Tm*_. An early study showed that *E. coli* catalyzes C2 thiolation of cytosine in tRNA using an enzyme that contains an essential, but oxygen labile, cluster (33). A mechanism involving sulfur transfer of a noncubane sulfide ligand was postulated but not demonstrated. A report describing enzymes used for synthesis of tRNA containing 2-thiouridine or 4-thiouridine in *Methanococcus maripaludis* and of 2-thiouridine in eukaryotes suggested they possess [3Fe-4S] clusters (34), but later a case was made that these enzymes contain oxygen-labile [4Fe-4S] clusters (11). The 4-thiouridine tRNA synthase from *M. maripaludis* uses exogenous sulfide as a sulfur source (34), consistent with an extra cubane-associated sulfide. Synthesis of 5-methyl-2-thiouridine in the tRNA of *Thermus thermophilus* involves two proteins: TtuA contains a PP-loop domain for activating the substrate and an oxygen-labile [4Fe-4S] cluster, while TtuB is a sulfur transfer protein with a thiocarboxylate C terminus (28). The structure of the TtuA holoprotein in the presence of an ATP analog confirmed the presence of a [4Fe-4S] cluster bound by three cysteine residues and demonstrated the nucleotide was positioned nearby. Furthermore, the structure of a TtuA–TtuB complex revealed the close juxtaposition of the TtuB thiocarboxylate with the open coordination site on the [4Fe-4S] cluster (27). These authors proposed a model in which the TtuB thiocarboxylate donates its sulfide to the [4Fe-4S] cluster, with the noncuboidal sulfide then attacking the activated substrate. Direct structural evidence for an extra-cubane sulfide bound to a [4Fe-4S] cluster was obtained for TtuA from *Pyrococcus horikoshii* (26). That enzyme uses inorganic sulfide (abundant in this microorganism’s environment) as the sulfur source, rather than thiocarboxylated TtuB. Similar reactions are also likely to be used by TtuA from *T. maritima* (26), 2-thiouridine synthases of *T. thermophilus* (14) and *E. coli* (35), and probably by a broad range of other related enzymes (11, 14). Overall, the thionucleotide biosynthesis literature provides important precedents for the proposed mechanism of LarE_*Tm*_ involving interconversions between [4Fe-4S] and [4Fe-5S] states.

## Experimental procedures

### Vector construction and mutagenesis

Plasmid pET:LarE encoding the C-terminal His_6_-tagged form of LarE from *T. maritima* was obtained from Prof. Tom Desmet. Codon-optimized *larE* was synthesized by GeneArt Gene Synthesis (Thermo Fisher Scientific) and subcloned into the pET21 vector at the NdeI and XhoI restriction sites. Site-directed mutagenesis was carried out using the gap-repair method (36) with mutations confirmed by sequence analysis. The pET:LarE plasmid and its derivatives were transformed into *E. coli* BL21 (DE3). The strains, primers, and plasmids used in this study are summarized in Table 1. The C172A/C92A, C172A/C95A, and C172A/C92A/C95A variants were created using the C172A plasmid with the C92A, C95A, and C92A/95A forward and reverse primers, respectively.

**Table 1:** Strains, plasmids, and primers used in this study

Strain, plasmid, or primer	Characteristics or sequences	Description
Strains		
*E. coli*		
DH5α	F– φ80lacZΔ M15 Δ (*lacZYA-argF*) *U169 recA1 endA1 hsdR17* (rK– mK+) *phoA supE44* λ- *thi–1 gyrA96 relA1*	Thermo Fisher
BL21 (DE3)	fhuA2 [lon] ompT gal (λ DE3) [dcm] ΔhsdS λ DE3 = λ sBamHIo ΔEcoRI-B int::(lacI::PlacUV5::T7 gene1) i21 Δnin5	NEB
Plasmids		
pET:LarE	Amp^r^, pET21b with a 0.83-kb insert containing *larE* translationally fused to DNA encoding the 6x-His tag	
Primers		
LarE C92A-F	CCGCCTGATCGTGCCTATCTGTGTCGTAAACTGCGT	Gap Repair
LarE C92A-R	ACGATCAGGCGGATTTTCAATCAG	Gap Repair
LarE C95A-F	GATCGTTGTTATCTGGCCCGTAAACTGCGTGATAAC	Gap Repair
LarE C95A-R	CAGATAACAACGATCAGGCGG	Gap Repair
LarE C172A-F	GGATAAACCGGCAATGGCAGCCCTGTGTAGCCGTTTT	Gap Repair
LarE C172A-R	TGCCATTGCCGGTTTATCCCAGG	Gap Repair
LarE C174A-F	CCGGCAATGGCATGTCTGGCCAGCCGTTTT	Gap Repair
LarE C174A-R	CAGACATGCCATTGCCGGTTTATCCC	Gap Repair
LarE C172A/-C174A-F	GGATAAACCGGCAATGGCAGCCCTGGCCAGCCGTTTT	Gap Repair
LarE C172A/-C174A-R	CAGGGCTGCCATTGCCGGTTTATCCCAGG	Gap Repair
LarE C92A/-C95A-F	CCGCCTGATCGTGCCTATCTGGCCCGTAAACTGCGT	Gap Repair
LarE C92A/-C95A-R	ACGATCAGGCGGATTTTCAATCAG	Gap Repair

### LarE*_Tm_* purification

For aerobic purification of His_6_-tagged LarE_*Tm*_, *E. coli* BL21 (DE3) [pET:LarE] cells were grown in LB–Lennox medium supplemented with 100 mg/L carbenicillin at 37 °C with shaking at 225 RPM until reaching an OD_600_ of 0.5 to 1.0. The cells were induced with 0.4 mM IPTG and growth was continued for another 3 h at 37 °C with shaking. Cultures were harvested by centrifugation at 11,000*g* for 10 min, and cell pellets were stored at −80 °C until further use. The *E. coli* cell pellets were resuspended in 100 mM Tris–HCl, pH 7.2, buffer containing 300 mM NaCl, 5 to 10 mM 2-mercaptoethanol (βME), 2 mM phenylmethylsulfonyl fluoride, and 1 U/ml of Benzonase (EMD). The suspended cells were lysed using a French pressure cell at 16,000 psi and 4 °C. Cell-free lysates were obtained by centrifugation at 100,000*g* for 1 h and 4 °C. The lysates were loaded onto HisPur nickel-nitrilotriacetic acid resin (Thermo Fisher) that was equilibrated in 100 mM Tris–HCl, pH 7.2, buffer containing 300 mM NaCl using gravity flow at 4 °C. His_6_-tagged LarE_*Tm*_ was eluted from the column by stepwise increases in the concentration of imidazole (5, 20, 50, 100, then 350 mM imidazole) in some cases containing 5 to 10 mM βME. The homogeneity of the purified protein was checked by 12% SDS-PAGE. Protein concentrations were measured based on the absorbance at 280 nm (the His_6_-tagged LarE_*Tm*_ subunit has a calculated extinction coefficient at 280 nm of 24,200 M^-1^ cm^-1^) (https://web.expasy.org/protparam/) or by using the Bradford reagent (Bio-Rad). The variants of His_6_-tagged LarE_*Tm*_ were purified using the same conditions as those for the WT enzyme.

Anoxic samples of His_6_-tagged LarE_*Tm*_ were purified from 0.3 l of aerobically grown *E. coli* cultures supplemented with 200 μM ammonium ferric citrate and 25 μM L-methionine (37) within an anaerobic chamber (Coy Laboratory Products) that contained an atmosphere of 95% N_2_ plus 5% H_2_ (<2 ppm [O_2_]) and a palladium catalyst. The cells were lysed by addition of 0.2 to 0.3 mg/ml lysozyme, followed by incubation at 37 ⁰C for 1 h. Cell-free lysates were obtained by centrifugation at 13,800*g* for 1 h at RT. Chromatography of the lysates was performed as described previously but inside the chamber at RT. All buffers used for the purification were degassed and allowed to equilibrate in the anaerobic atmosphere for at least 3 days prior to use.

For anaerobic growth, *E. coli* cells were cultivated in LB broth (Miller medium from NEOGEN Culture Media) supplemented with 0.5% w/v glucose, 100 mM Mops/NaOH, pH 7.4, 50 μg/ml carbenicillin, and 2 mM ferric ammonium citrate. The cultures were first grown aerobically at 25 ⁰C until reaching an OD_600_ of ∼0.7, then bubbled with Ar gas for 15 min and immediately moved into the anaerobic chamber. The anoxic medium for each sample was adjusted to contain 2 mM L-methionine, 2 mM L-cysteine, 25 mM sodium fumarate, and 0.5 mM IPTG (38). The containers were tightly sealed with parafilm and closed with GL-45 Pyrex caps. The cultures were grown anaerobically at 25 ⁰C with minimum or no shaking for another 16 to 24 h, returned to the anaerobic chamber, transferred into centrifugal tubes, and harvested by centrifugation. The cell pellets were disrupted and LarE was purified inside the anaerobic chamber as mentioned previously

### Assembly of the LarE_*Tm*_ iron–sulfur cluster

Using anoxic buffer solutions within the anaerobic glove box, samples of His_6_-tagged LarE_*Tm*_ (previously purified using aerobic or anaerobic conditions) were incubated for 10 min at RT with DTT (at 50-fold concentration over the enzyme), followed by addition of 10 mM L-cysteine and His_6_-tagged IscS_*Ec*_ (used at 1/50^th^ the amount of LarE_*Tm*_), then incubated for 20 min. Subsequently, a 10-fold molar excess of FeCl_3_ was slowly added, and the samples were incubated for 3 h at RT. Excess FeCl_3_ and L-cysteine were removed using PD-10 desalting columns (GE Healthcare) that were equilibrated with 100 mM Tris–HCl, pH 7.2, buffer containing 300 mM NaCl. For chemical assembly of the iron-sulfur cluster, His_6_-tagged LarE_*Tm*_ was incubated with a 50-fold molar excess of DTT for 1 h, then slowly adjusted to have a 10-fold molar excess of FeCl_3_, followed by addition of a 10-fold molar excess of freshly prepared Na_2_S, and incubated for another 3 h inside the anaerobic chamber. The excess reagents for both methods were removed by passing the mixtures through PD MiniTrap G-25 columns (GE Healthcare) that were pre-equilibrated with 100 mM Tris–HCl (pH 7.2) buffer containing 0.3 M NaCl. The resulting LarE_*Tm*_ holoprotein samples were concentrated using 10 kDa cutoff Amicon Ultra filters (Merck–Millipore) at RT. All of the samples were stored at RT and kept anoxic in an anaerobic glove box.

### Purification of additional proteins

LarA, LarB, LarE, and LarC from *L. plantarum* (LarA_*Lp*_, LarB_*Lp*_, LarE_*Lp*_, and LarC_*Lp*_, respectively), LarA_*Tt*_ apoprotein, and His_6_-tagged IscS_*Ec*_ were purified as previously described (4, 6, 7, 8, 10, 39).

### Physical characterization of LarE_*Tm*_ and IscS_*Ec*_

To determine the subunit sizes of His_6_-tagged LarE*_Tm_* and IscS_*Ec*_ samples and to examine the sulfane sulfur content of IscS_*Ec*_, protein samples (10–20 μM depending on the experiment) were injected onto a cyano-chemistry HPLC column that was equilibrated in 0.1% formic acid and eluted with an increasing gradient of acetonitrile. The fractions were analyzed by ESI-MS using a XEVO G2-XS instrument in positive ionization mode. The protein masses were derived from the MS data using MaxEnt (Waters Corp).

The native size of His_6_-tagged LarE_*Tm*_ was determined by size-exclusion chromatography using a Superdex 200 Increase 10/300 GL column equilibrated in 100 mM Tris–HCl, pH 7.2, buffer containing 300 mM NaCl at a flow rate of 0.5 ml/min, followed by particulate removal using a 0.2 μm filter that was placed in line with a miniDAWN TREOS multiangle light scattering detector and T-rEX refractive index meter (Wyatt Technology). The data were processed using ASTRA (Wyatt).

Native nano–ESI-MS of LarE_*Tm*_ was performed using a Q Exactive UHMR instrument with a flow of nitrogen gas inside the sample holder so that samples were maintained under an anoxic atmosphere during the experiment. This was achieved by setting the sheath gas, auxiliary gas flow, and sweep gas flow to 4 (arbitrary units). Other instrumental parameters include capillary temperature of 250 °C, resolution of 12,500, C-trap pressure of 2, ion transfer *m*/*z* optimization set to high *m*/*z*, in-source trapping set to on, desolvation voltage of −150 V, detector *m*/*z* optimization set to low *m*/*z*, extended trapping of 50 eV, and, when optimizing monomeric species, applying an in-source CID of 100 eV. Data were collected over a broad *m*/*z* range (500–4000 *m*/*z*). Signals of sufficient signal-to-noise ratio were identified based on LarE mass calculations (Tables S1 and S2) accounting for an expected mass measurement accuracy of 10 ppm. Furthermore, recorded isotope abundance profiles were compared to theoretical abundance profiles for Fe- and S-containing protein ions. Unidentified signals in our native MS likely correspond to buffer component LarE_*Tm*_ adducts commonly observed in such data (40, 41).

### LarE_*Tm*_ enzyme activity

Two methods were used to assay the activity of His_6_-tagged LarE_*Tm*_. First, a previously described LC-ESI-MS procedure (5) was used to monitor (i) the loss of P2CMN (*m*/*z* = 378 eluting at 4.62 min), (ii) the generation P2TMN (*m*/*z* = 410, eluting at 4.94 min), and (iii) the intermediate PCTMN (*m*/*z* = 394, eluting after 4.78 min). Secondly, we used an indirect coupled assay that relied on the generation of Lar activity (5). LarB*_Lp_* (10 μm) was first mixed with NaAD (0.2 mM), ATP (2 mM), MgCl_2_ (20 mM), and NaHCO_3_ (50 mM) in Tris–HCl buffer (100 mm, pH 7) and incubated for at least 10 to 15 min. The resulting solution (containing P2CMN) was mixed with anoxic samples of His_6_-tagged LarE_*Tm*_ (degassed and maintained inside the anaerobic chamber for at least 1 day; variable concentrations were used depending on the experiment), incubated for the times indicated at RT, then heat treated at 80 °C for 10 min. The sample was centrifuged (20,000*g* for 5 min), and the supernatant (containing P2TMN) was incubated with an equal volume of a mixture of LarC*_Lp_* (2.5 μM), MgCl_2_ (10 mM), βME (10 mM), and CTP (0.1 mM) in Mes buffer (100 mM, pH 6) for 1 h at RT. The reaction was stopped by heat treatment at 80 °C for 10 to 20 min followed by centrifugation (20,000*g* for 10 min). The solution (containing the synthesized NPN) was mixed with an equal volume of buffer containing L-lactate (45 mM) and LarA_*Tt*_ apoprotein (0.8 μM) in Hepes buffer (100 mM, pH 7). The solution was incubated for 5 min at 50 °C and then stopped by heat treatment at 90 °C using a thermocycler. The resulting D-lactate concentration was measured using a D-lactic acid/L-lactic acid commercial test (Megazyme), as described previously (4). The absorbance of NADH was monitored at 340 nm with a Shimadzu spectrophotometer (model UV 2600).

### Biophysical characterization of the metallocenter

The metal content of His_6_-tagged LarE_*Tm*_ was assessed by using ICP-OES. Samples were prepared by boiling for 1 h in 35% (w/v) nitric acid before analysis using a model 710 Varian ICP-OES. The amount of acid labile sulfide was determined as previously described (42), with all steps carried out inside of an anaerobic chamber.

The UV-vis spectra were recorded under anaerobic conditions using an Ocean Optics UV-vis spectrophotometer or with a Shimadzu spectrophotometer (model UV 2600) under aerobic conditions. The effect of oxygen on the sample was investigated by monitoring changes in the spectrum over time after aerobic exposure. To examine the effects of reductant on the chromophore, protein samples (in 100 mM Tris, pH 7.2, buffer containing 300 mM NaCl) were mixed in anaerobic conditions with at least a 10-fold molar excess of sodium dithionite and incubated for 5 to 10 min before recording the spectra.

For EPR analysis, His_6_-tagged LarE_*Tm*_ was purified from anaerobically grown cells, its cluster assembled, the protein concentrated up to 600 μM and adjusted to contain 10% glycerol, then frozen in liquid nitrogen. EPR data were collected on a Bruker E680X EPR spectrometer operating at X-band and equipped with a Bruker SHQ-E cavity. The sample temperature was poised at 10 K using an Oxford ESR-900 liquid helium cryostat and an ITC-503 temperature controller.

## Data availability

All data are available within the article and its supporting information file. The data underlying this article will be shared on a reasonable basis by submitting a request to the corresponding author.

## Supporting information

This article contains supporting information (43).

## Conflict of interest

The authors declare that they have no conflicts of interest with the contents of this article.
