# Supplementary material for: Characterization of a [4Fe-4S]-dependent LarE sulfur insertase that facilitates nickel-pincer nucleotide cofactor biosynthesis in Thermotoga maritima
Source: J Biol Chem. 2022 Jun 11;298(7):102131. doi: 10.1016/j.jbc.2022.102131 (PMC9283937; doi:10.1016/j.jbc.2022.102131)
Supplement: Supplemental information [file mmc1.pdf]

Supporting Information for

Characterization of a [4Fe-4S]-Dependent LarE Sulfur Insertase that Facilitates Nickel-Pincer Nucleotide Cofactor Biosynthesis in *Thermotoga maritima*

Shramana Chatterjee[1], Kristine F. Parson[2], Brandon T. Ruotolo[2], John McCracken[3], Jian Hu[3,4], and Robert P. Hausinger[1,4,*]

From the [1]Department of Microbiology and Molecular Genetics, Michigan State University, East Lansing, Michigan, USA; [2]Department of Chemistry, University of Michigan, Ann Arbor, Michigan, USA; [3]Department of Chemistry, Michigan State University, East Lansing, Michigan, USA; and [4]Department of Biochemistry and Molecular Biology, Michigan State University, East Lansing, Michigan, USA

Running Title: [4Fe-4S]-dependent sulfur-transferase LarE

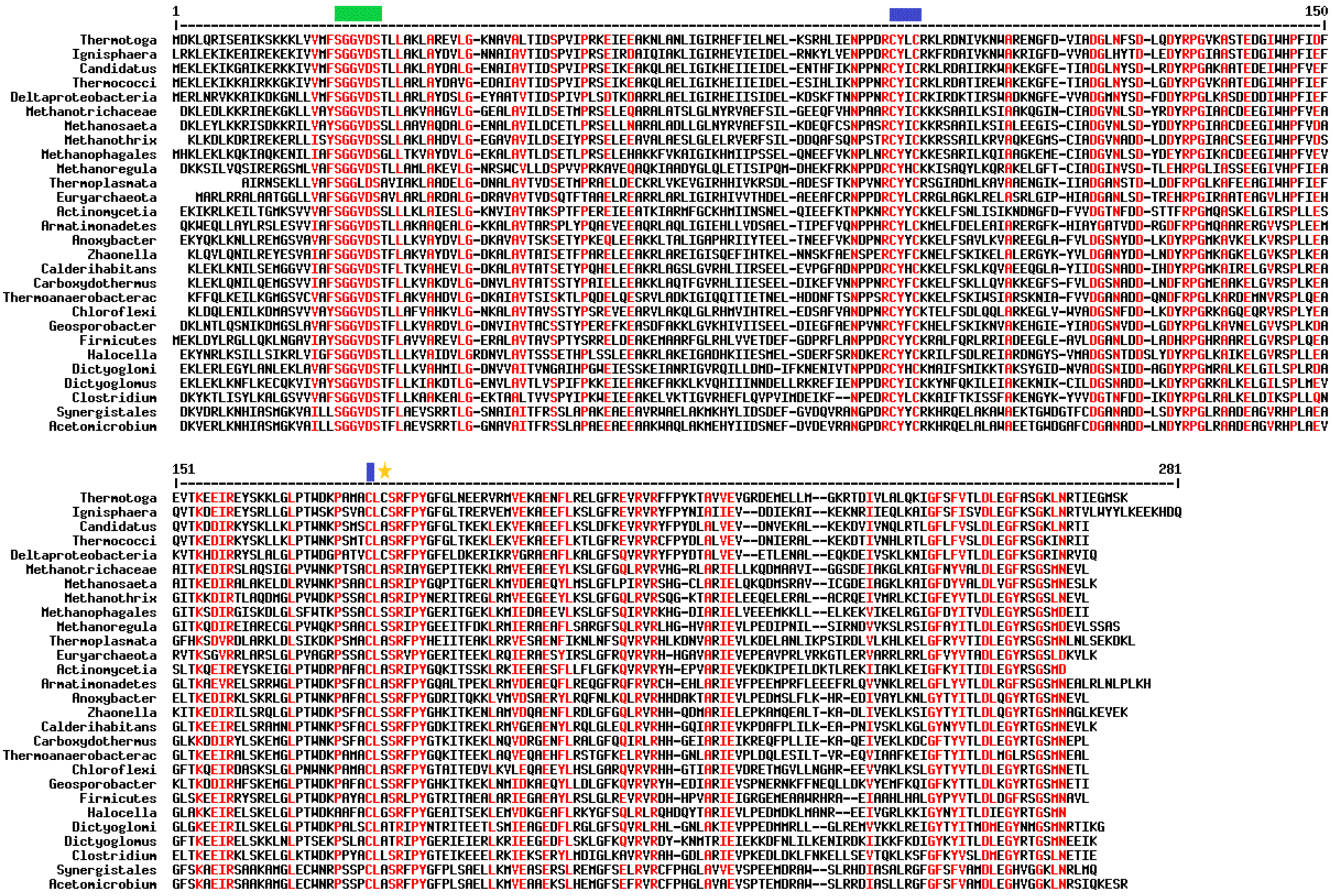
1
150
Thermotoga
Ignisphaera
Candidatus
Thermococci
Deltaproteobacteria
Methanotrichaceae
Methanosaeta
Methanothrix
Methanophagales
Methanoregula
Thermoplasmata
Euryarchaeota
Actinomycetia
Armatimonadetes
Anoxybacter
Zhaonella
Calderihabitans
Carboxydothermus
Thermoanaerobacterac
Chloroflexi
Geosporobacter
Firmicutes
Halocella
Dictyoglomi
Dictyoglomus
Clostridium
Synergistales
Acetomicrobium
151
281

Figure S1. Sequence comparison of LarE$_{Tm}$ to selected other LarE sequences. The sequences were chosen from those identified by BLAST using the non-redundant GenBank coding sequence translations with E-scores below e-73. Full species names for the genera shown are: *Thermotoga maritima*, *Ignisphaera aggregans*, *Candidatus Hydrothermae bacterium*, *Thermococci archaeon*, *Deltaproteobacteria bacterium*, *Methanotrichaceae archaeon*, *Methanosaeta sp.* PtaU1.Bin112, *Methanothrix soehngenii GP6*, *Methanophagales archaeon, Methanoregula formicica*, *Thermoplasmata archaeon*, *Euryarchaeota archaeon*, *Actinomycetia bacterium*, *Armatimonadetes bacterium*, *Anoxybacter fermentans*, *Zhaonella formicivorans*, *Calderihabitans maritimus*, *Carboxydothermus hydrogenoformans*, *Thermoanaerobacteraceae bacterium*, *Chloroflexi bacterium*, *Geosporobacter subterraneus*, *Firmicutes bacterium*, *Halocella sp.* SP3-1, *Dictyoglomi bacterium*, *Dictyoglomus thermophilum*, *Clostridium homopropionicum*, *Synergistales bacterium* 54_24, *Acetomicrobium sp.* S15 (DSM 107314). The sequence alignment was performed using *Multalin* (43). Identities are shown in red font. An SGGXDS motif associated with PP-loop pyrophosphatase family members is indicated by the green bar. Three cysteines associated with a CXXC-C motif are shown by the two blue segments. A fourth cysteine present in three sequences is shown by the yellow star.

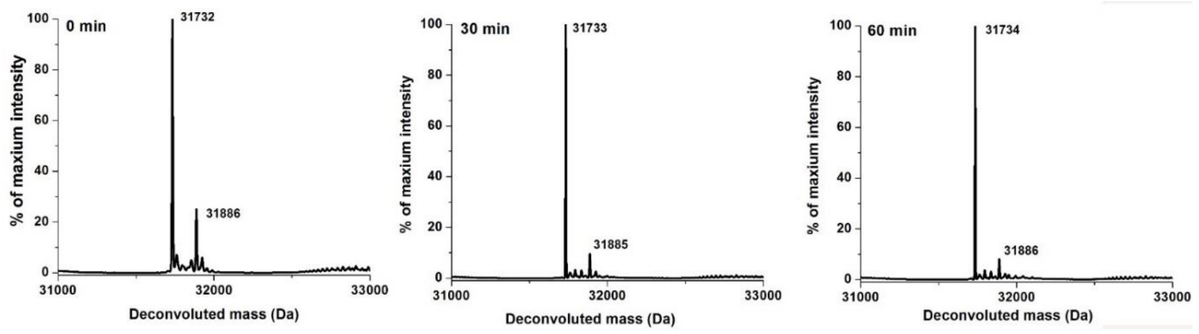

Figure S2. Mass spectrometric analysis reveals a lack of change for LarE$_{Tm}$ during P2TMN synthesis. The His_6-tagged LarE subunit lacking its amino-terminal methionine residue remains unchanged in size when comparing samples that were incubated with P2CMN, 20 mM $MgCl_2$, and 2 mM ATP for 0, 30, and 60 min. The small peak at *m*/*z* 31,186 likely represents His_6-tagged LarE in a mixed disulfide linkage with βME.

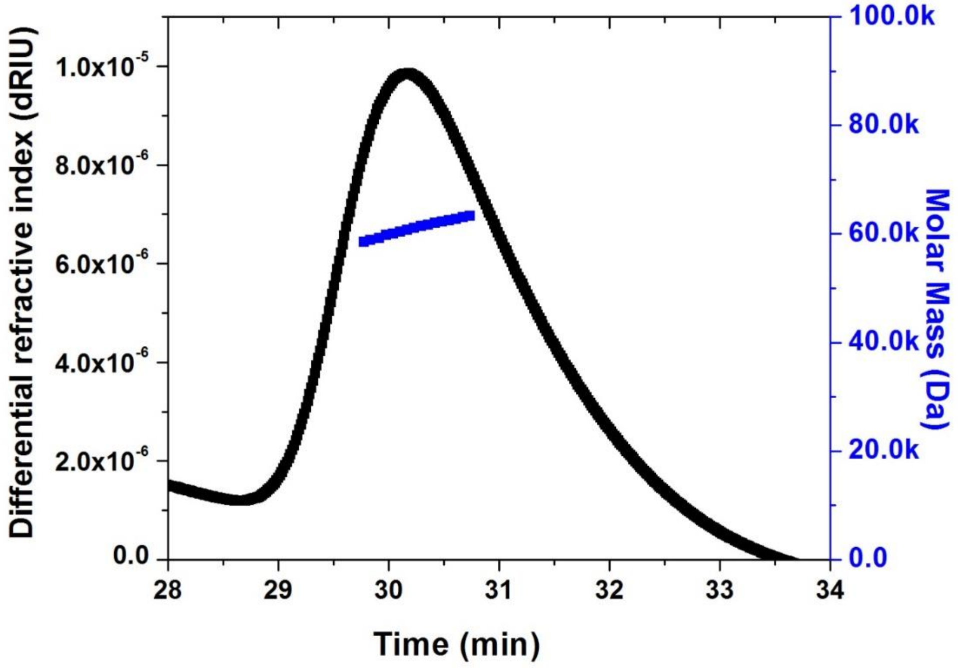

Figure S3: SEC MALS analysis of His_6-tagged LarE$_{Tm}$. The results indicate a dimeric quaternary structure.

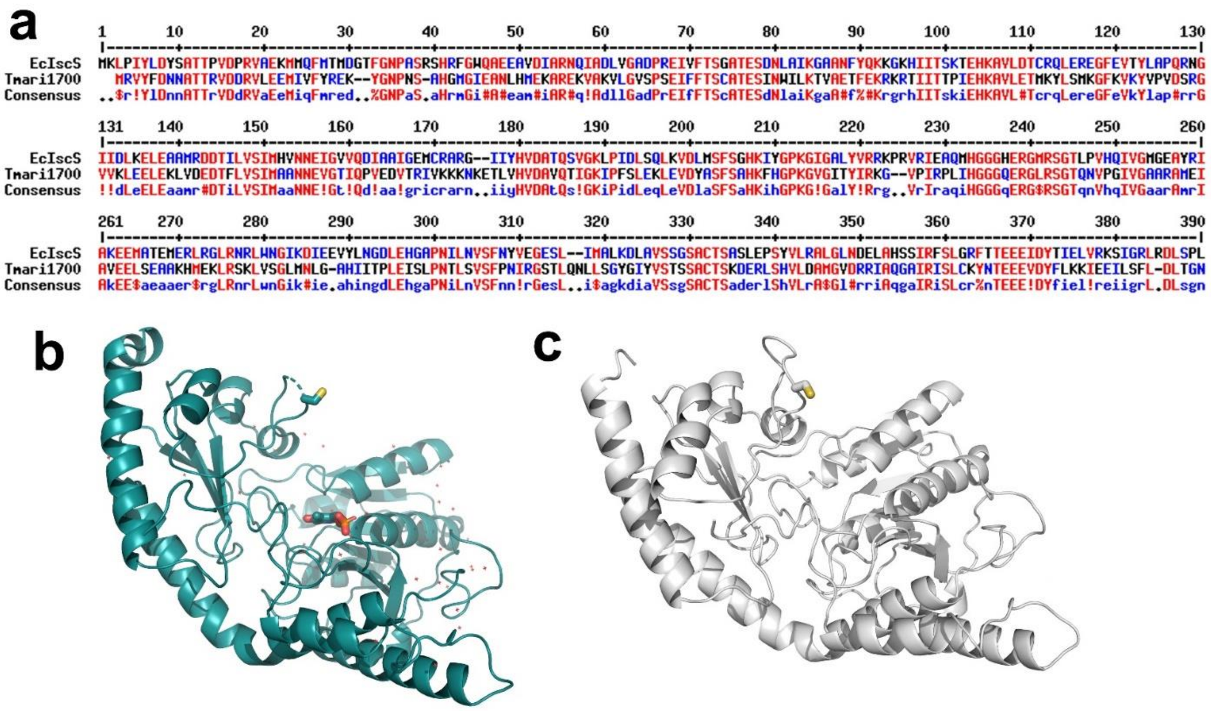

Figure S4. Comparison of cysteine desulfurase sequences and structures from *E. coli* (IscS$_{Ec}$) and one of two related proteins in *T. maritima* (IscS$_{Tm}$). (a). Sequence alignment. (b) Crystal structure of IscS$_{Ec}$ (PDB: 3LVK) including the bound pyridoxal phosphate (space fill depiction) and showing the cysteine residue (stick view) that forms a persulfide. (c) Homology model of IscS$_{Tm}$ depicting the conserved cysteine residue (stick view).

Table S1

Native mass spectrometry peak list for the 12+ monomeric species of LarE$_{Tm}$[a]

12+ Monomer (31,738 Daltons)			
LarE$_{Tm}$ Monomer + Species	Theoretical *m/z*	Actual (peak) *m/z*	Difference *(m/z)*
Na^{++}	2634.8	2635.1	0.3
Fe	2637.5	2637.5	0.0
[Fe-S]	2640.2	2640.3	0.1
[Fe-2S]	2642.8	2642.8	0.0
[2Fe-S]	2644.8	2645.7	0.9
[2Fe-2S]	2647.5	2648.3	0.8
[2Fe-3S]	2650.2	2650.9	0.7
[3Fe-3S]	2654.8	2654.0	0.8
[3Fe-4S]	2657.5	2657.3	0.2
[4Fe-4S]	2662.2	2661.9	0.3

[a] For each species shown, the numbers within the brackets represent the total number of iron and sulfur atoms associated with the LarE$_{Tm}$ monomer subunit and do not necessarily equate to the type of bound iron-sulfur cluster. The masses tabulated include the theoretical *m*/*z*, the actual (peak) *m*/*z*, and the calculated difference, with values near zero providing confidence in the iron and sulfur content assignments.

Table S2

Native mass spectrometry peak list for the 15+ dimeric species of LarE$_{Tm}$[a] including theoretical *m*/*z*, the actual (peak) *m*/*z*, and calculated difference, illustrating the confidence in the iron and sulfur content assignments.

15+ Dimer (63,476 Daltons)			
LarE$_{Tm}$ Dimer + Species	Theoretical *m/z*	Actual (peak) *m/z*	Difference (*m/z*)
[3Fe-4S][3Fe-4S]	4256.2	4257.4	1.2
[4Fe-4S][4Fe-4S]	4263.7	4262.6	1.1
[4Fe-5S][4Fe-5S]	4265.8	4264.8	1.0
[4Fe-5S][5Fe-5S]	4271.7	4273.1	1.4
[5Fe-5S][5Fe-5S]	4275.4	4275.5	0.1

[a] For each species shown, the numbers within the brackets represent the total number of iron and sulfur atoms associated with each LarE$_{Tm}$ subunit in the dimer and do not necessarily equate to the type of bound iron-sulfur cluster. The masses tabulated include the theoretical *m*/*z*, the actual (peak) *m*/*z*, and the calculated difference, with values near zero providing confidence in the iron and sulfur content assignments.

References:

CORPET, F. (1988) Multiple sequence alignment with hierarchical clustering. *Nucl. Acids Res.* **16** (22), 10881-10890
